# Diagnosis and management of pseudohypoparathyroidism and related disorders: first international Consensus Statement

**DOI:** 10.1038/s41574-018-0042-0

**Published:** 2018-06-29

**Authors:** Giovanna Mantovani, Murat Bastepe, David Monk, Luisa de Sanctis, Susanne Thiele, Alessia Usardi, S. Faisal Ahmed, Roberto Bufo, Timothée Choplin, Gianpaolo De Filippo, Guillemette Devernois, Thomas Eggermann, Francesca M. Elli, Kathleen Freson, Aurora García Ramirez, Emily L. Germain-Lee, Lionel Groussin, Neveen Hamdy, Patrick Hanna, Olaf Hiort, Harald Jüppner, Peter Kamenický, Nina Knight, Marie-Laure Kottler, Elvire Le Norcy, Beatriz Lecumberri, Michael A. Levine, Outi Mäkitie, Regina Martin, Gabriel Ángel Martos-Moreno, Masanori Minagawa, Philip Murray, Arrate Pereda, Robert Pignolo, Lars Rejnmark, Rebecca Rodado, Anya Rothenbuhler, Vrinda Saraff, Ashley H. Shoemaker, Eileen M. Shore, Caroline Silve, Serap Turan, Philip Woods, M. Carola Zillikens, Guiomar Perez de Nanclares, Agnès Linglart

**Affiliations:** 1 Fondazione IRCCS Ca’ Granda Ospedale Maggiore Policlinico, Endocrinology Unit, Department of Clinical Sciences and Community Health, University of Milan, Milan, Italy; 2 Endocrine Unit, Department of Medicine, Massachusetts General Hospital and Harvard Medical School, Boston, MA USA; 3 Imprinting and Cancer Group, Cancer Epigenetic and Biology Program (PEBC), Institut d’Investigació Biomedica de Bellvitge (IDIBELL), Barcelona, Spain; 4 Pediatric Endocrinology Unit, Department of Public Health and Pediatric Sciences, University of Torino, Turin, Italy; 5 Division of Pediatric Endocrinology and Diabetes, Department of Pediatrics, University of Lübeck, Lübeck, Germany; 6 APHP, Reference Center for Rare Disorders of Calcium and Phosphate Metabolism, Platform of Expertise Paris-Sud for Rare Diseases and Filière OSCAR, Bicêtre Paris Sud Hospital (HUPS), Le Kremlin-Bicêtre, France; 7 APHP, Endocrinology and diabetes for children, Bicêtre Paris Sud Hospital (HUPS), Le Kremlin-Bicêtre, France; 8 Developmental Endocrinology Research Group, School of Medicine, Dentistry and Nursing, University of Glasgow, Glasgow, UK; 9 IPOHA, Italian Progressive Osseous Heteroplasia Association, Cerignola, Foggia Italy; 10 K20, French PHP and related disorders patient association, Jouars Pontchartrain, France; 11 APHP, Department of medicine for adolescents, Bicêtre Paris Sud Hospital (HUPS), Le Kremlin-Bicêtre, France; 12 Institute of Human Genetics, Medical Faculty, RWTH Aachen University, Aachen, Germany; 13 Department of Cardiovascular Sciences, Center for Molecular and Vascular Biology, Gasthuisberg, University of Leuven, Leuven, Belgium; 14 AEPHP, Spanish PHP and related disorders patient association, Huércal-Overa, Almería, Spain; 15 Albright Center & Center for Rare Bone Disorders, Division of Pediatric Endocrinology & Diabetes, Connecticut Children’s Medical Center, Farmington, CT USA; 16 Department of Pediatrics, University of Connecticut School of Medicine, Farmington, CT USA; 17 APHP, Department of Endocrinology, Cochin Hospital (HUPC), Paris, France; 18 University of Paris Descartes, Sorbonne Paris Cité, Paris, France; 19 Department of Medicine, Division of Endocrinology and Centre for Bone Quality, Leiden University Medical Center, Leiden, Netherlands; 20 INSERM U1169, Bicêtre Paris Sud, Paris Sud – Paris Saclay University, Le Kremlin-Bicêtre, France; 21 APHP, Department of Endocrinology and Reproductive Diseases, Bicêtre Paris Sud Hospital (HUPS), Le Kremlin-Bicêtre, France; 22 INSERM U1185, Paris Sud – Paris Saclay University, Le Kremlin-Bicêtre, France; 23 UK acrodysostosis patients’ group, London, UK; 24 Department of Genetics, Reference Centre for Rare Disorders of Calcium and Phosphate Metabolism, Caen University Hospital, Caen, France; 25 BIOTARGEN, UNICAEN, Normandie University, Caen, France; 26 APHP, Department of Odontology, Bretonneau Hospital (PNVS), Paris, France; 27 Department of Endocrinology and Nutrition, La Paz University Hospital, Madrid, Spain; 28 Department of Medicine, Autonomous University of Madrid (UAM), Madrid, Spain; 29 Endocrine Diseases Research Group, Hospital La Paz Institute for Health Research (IdiPAZ), Madrid, Spain; 30 Division of Endocrinology and Diabetes and Center for Bone Health, Children’s Hospital of Philadelphia and Department of Pediatrics, University of Pennsylvania Perelman School of Medicine, Philadelphia, PA USA; 31 Children’s Hospital, University of Helsinki and Helsinki University Hospital, Helsinki, Finland; 32 Osteometabolic Disorders Unit, Hormone and Molecular Genetics Laboratory (LIM/42), Endocrinology Division, Hospital das Clínicas HCFMUSP, Faculty of Medicine, University of Sao Paulo, Sao Paulo, Brazil; 33 Department of Endocrinology, Hospital Infantil Universitario Niño Jesús, CIBERobn, ISCIII, Madrid, Spain; 34 Department of Pediatrics, Autonomous University of Madrid (UAM), Madrid, Spain; 35 Endocrine Diseases Research Group, Hospital La Princesa Institute for Health Research (IIS La Princesa), Madrid, Spain; 36 Division of Endocrinology, Chiba Children’s Hospital, Chiba, Japan; 37 Department of Paediatric Endocrinology, Royal Manchester Children’s Hospital, Manchester University NHS Foundation Trust, Manchester, UK; 38 Molecular (Epi)Genetics Laboratory, BioAraba National Health Institute, Hospital Universitario Araba-Txagorritxu, Vitoria-Gasteiz, Alava Spain; 39 Department of Medicine, Mayo Clinic, Rochester, MN USA; 40 Department of Endocrinology and Internal Medicine, Aarhus University Hospital, Aarhus, Denmark; 41 Department of Endocrinology and Diabetes, Birmingham Children’s Hospital, Birmingham, UK; 42 Pediatric Endocrinology and Diabetes, Vanderbilt University Medical Center, Nashville, TN USA; 43 Departments of Orthopaedic Surgery and Genetics, Center for Research in FOP and Related Disorders, Perelman School of Medicine, University of Pennsylvania, Philadelphia, PA USA; 44 APHP, Service de Biochimie et Génétique Moléculaires, Hôpital Cochin, Paris, France; 45 Department of Pediatrics, Division of Endocrinology and Diabetes, Marmara University, Istanbul, Turkey; 46 Department of Internal Medicine, Bone Center Erasmus MC – University Medical Center Rotterdam, Rotterdam, Netherlands

**Keywords:** Parathyroid diseases, Diagnosis, Therapeutics

## Abstract

This Consensus Statement covers recommendations for the diagnosis and management of patients with pseudohypoparathyroidism (PHP) and related disorders, which comprise metabolic disorders characterized by physical findings that variably include short bones, short stature, a stocky build, early-onset obesity and ectopic ossifications, as well as endocrine defects that often include resistance to parathyroid hormone (PTH) and TSH. The presentation and severity of PHP and its related disorders vary between affected individuals with considerable clinical and molecular overlap between the different types. A specific diagnosis is often delayed owing to lack of recognition of the syndrome and associated features. The participants in this Consensus Statement agreed that the diagnosis of PHP should be based on major criteria, including resistance to PTH, ectopic ossifications, brachydactyly and early-onset obesity. The clinical and laboratory diagnosis should be confirmed by a molecular genetic analysis. Patients should be screened at diagnosis and during follow-up for specific features, such as PTH resistance, TSH resistance, growth hormone deficiency, hypogonadism, skeletal deformities, oral health, weight gain, glucose intolerance or type 2 diabetes mellitus, and hypertension, as well as subcutaneous and/or deeper ectopic ossifications and neurocognitive impairment. Overall, a coordinated and multidisciplinary approach from infancy through adulthood, including a transition programme, should help us to improve the care of patients affected by these disorders.

## Abstract (web summary)

This Consensus Statement gives recommendations for the diagnosis and management of patients with pseudohypoparathyroidism and related disorders. A coordinated and multidisciplinary approach from infancy through adulthood is recommended to improve the care of these patients.

## Introduction

**Fig. 1:**
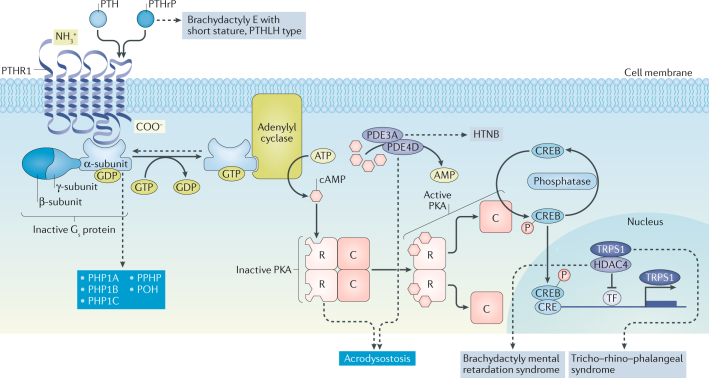
Molecular defects in the PTH–PTHrP signalling pathway in PHP and related disorders. Upon ligand binding (parathyroid hormone (PTH) and parathyroid hormone-related protein (PTHrP) are shown on the figure), the G protein coupled PTH/PTHrP receptor type 1 (PTHR1) activates the heterotrimeric G_s_ protein. The G_s_α subunit triggers the activation of adenylyl cyclase, which leads to cAMP synthesis. cAMP then binds to the regulatory 1 A subunits (R) of protein kinase A (PKA), the predominant effector of cAMP. Upon cAMP binding, the catalytic subunits (C) dissociate from the R subunits and phosphorylate numerous target proteins, including cAMP-responsive binding elements (CREB) and the phosphodiesterases (PDEs; such as PDE3A and PDE4D). CREB activates the transcription of cAMP-responsive genes. Intracellular cAMP is then deactivated by PDEs, including PDE4D and PDE3A. The main clinical features of pseudohypoparathyroidism (PHP) and related disorders are due to molecular defects within the PTH–PTHrP signalling pathway, with the exception, perhaps, of ectopic ossification. The diseases caused by alterations in the genes that encode the indicated proteins are shown in blue boxes. Differential diagnoses are shown in grey boxes. CRE, cAMP response element; HDAC4, histone deacetylase 4; G protein, trimer α, β and γ; HTNB, autosomal dominant hypertension and brachydactyly type E syndrome; PHP, pseudohypoparathyroidism; PHP1A, pseudohypoparathyroidism type 1A; PHP1B, pseudohypoparathyroidism type 1B; PHP1C, pseudohypoparathyroidism type 1C; POH, progressive osseous heteroplasia; PPHP, pseudopseudohypoparathyroidism; PTHLH, parathyroid hormone-like hormone; TF, transcription factor; TRPS1, zinc-finger transcription factor TRPS1.

Pseudohypoparathyroidism (PHP) and related disorders are associated with a spectrum of abnormal physical characteristics as well as neurocognitive and endocrine abnormalities that are caused primarily by molecular defects that impair hormonal signalling via receptors that are coupled, through the α-subunit of the stimulatory G protein (G_s_α), to activation of adenylyl cyclase (Fig. 1).

The term PHP (Online Mendelian Inheritance in Man (OMIM) #103580 for PHP type 1A (PHP1A), #603233 for PHP type 1B (PHP1B) and #612462 for PHP type 1C (PHP1C)) describes disorders that share common biochemical features of hypoparathyroidism (that is, hypocalcaemia and hyperphosphataemia) that are the result of resistance of target tissues to the biological actions of parathyroid hormone (PTH). In some cases, resistance to other hormones (such as TSH, gonadotropins, growth hormone-releasing hormone (GHRH) and calcitonin) that have receptors coupled via G_s_α is observed. Patients with PHP1A and PHP1C are also characterized by the variable expression of a collection of physical features, termed Albright hereditary osteodystrophy (AHO), which includes premature closure of growth plates and short bones, short stature, a stocky build, ectopic ossifications and other poorly defined abnormalities. In some patients, the physical features of AHO might be present in the absence of hormone resistance. Furthermore, based on the number of AHO features and the extent of ectopic ossifications, patients might be classified as having pseudopseudohypoparathyroidism (PPHP; OMIM #612463), progressive osseous heteroplasia (POH; OMIM #166350) or osteoma cutis. PHP and PPHP were initially described by Fuller Albright and colleagues in 1942 (ref.^1^) and 1952 (ref.^2^), respectively; POH was reported more than five decades later, in 1994 (ref.^3^). Other features have been attributed to these disorders since their identification, such as intrauterine growth failure, early-onset obesity (that is, development in the first few months of life and full expression before the end of infancy), hypogonadism, growth hormone (GH) deficiency and cognitive impairment, including developmental delay and loss of intellectual function^4^. Acrodysostosis (OMIM #101800) refers to a group of chondrodysplasias that resemble PHP in some patients, owing to the presence of brachydactyly and often resistance to PTH and TSH, but differ from PHP owing to more extensive facial dysmorphism, nasal hypoplasia and often developmental delay^5,6^.

The exact prevalence of PHP is unknown. Studies published in 2000 and 2016 estimated the prevalence to be 0.34 in 100,000 in Japan^7^ and 1.1 in 100,000 in Denmark^8^. A limitation of both studies is that the investigators did not confirm the clinical diagnosis of PHP by a molecular analysis for the majority of the patients. The prevalence of POH has never been estimated. However, it seems to be extremely rare, as <60 cases have been reported worldwide up to December 2016 (refs^9,10^). The overlap between PPHP and PHP1A means that determining the prevalence of PPHP is even more complicated. Acrodysostosis is uncommon; the prevalence of the disease is unknown, and clinical, biochemical and radiological features overlap with those of PHP1A and PPHP^6,11–15^.

A molecular cause can be identified in an estimated 80–90% of patients with PHP or related disorders^16,17^. The most common underlying mechanisms are de novo or autosomal dominantly inherited genetic mutations and/or epigenetic, sporadic or genetic-based alterations, within or upstream of *GNAS*^4,18,19^, *PRKAR1A*^6^, *PDE4D*^5,20^ or *PDE3A*^21^ (Fig. 1; Supplementary Table 1).

In the absence of molecular analysis, the clinical and biochemical overlap between PHP and related disorders can lead to challenges in diagnostic classification and thus in understanding the natural history of the different types. A consensus meeting was organized to develop recommendations for the diagnosis and management of patients with PHP and related disorders.

The European Cooperation in Science and Technology (COST) action BM1208 (European Network for Human Congenital Imprinting Disorders, see Related links), the European Reference Network on Rare Endocrine Conditions (ENDO-ERN), the European Reference Network on Rare Bone Disorders (BOND-ERN), the European Calcified Tissue Society (ECTS), the Asian Pacific Paediatric Endocrine Society (APPES), the European Society of Human Genetics (ESHG), the Pediatric Endocrine Society (PES), the European Society of Endocrinology (ESE) and the European Society for Paediatric Endocrinology (ESPE) support this Consensus Statement.

## Methods

Thirty-seven participants from 13 countries were invited to participate in the development of this Consensus Statement based on their publication record and expertise in PHP and related disorders, and seven representatives from patient support groups from four countries were also included. Health-care professionals included endocrinologists, paediatric endocrinologists, paediatric nephrologists, a neonatologist, dentists, molecular biologists, molecular geneticists and a project coordinator/manager. Representatives from patient support groups (Spanish Association of Pseudohypopara-thyroidism (AEPHP), K20, the acrodysostosis group and the Italian Association of POH (IPOHA)) participated in all the discussions but did not vote for the final recommendations. Experts included representatives nominated by the council and clinical practice committees from six international societies, two European reference networks and a European network on imprinting disorders (COST BM1208). All participants signed a conflict of interest declaration, and the consensus was strictly supported by funding from academic or professional societies only, with no sponsorship from the pharmaceutical industry. A Delphi-like consensus methodology was adopted^22^.

A comprehensive literature search was conducted using PubMed including articles published from 1 January 1990 through 18 December 2016. The search terms used were “pseudohypoparathyroidism or PHP”, “Albright’s Hereditary Osteodystrophy or AHO”, “pseudopseudohypoparathyroidism or PPHP”, “progressive osseous heteroplasia or POH”, “acrodysostosis”, “osteoma cutis”, “ectopic ossifica-tions”, “subcutaneous ossifications”, “PTH resistance”, “brachydactyly”, “Fahr syndrome”, “calcaemia” and “hypocalcaemia”. The search was restricted to patients with a genetic diagnosis, so the following search terms were also included: “*GNAS* or *GNAS1* or Gs-alpha”, “*PRKAR1A”*, “*PDE4D*”, “*PDE3A*”, “*PTH1R* or *PTHR1*”, “*HOXD13*”, “*HDAC4*”, “*TRPS1*”, “*PTHLH*”, “multilocus hypomethylation or multilocus methylation defect or multilocus imprinting disturbance or MLID” and “prenatal testing and imprinting”. The year 1990 was chosen as a cut-off as it was the year in which the first inactivating *GNAS* mutations associated with PHP were described. Only publications in English were considered. Additional relevant articles on ini-tial clinical descriptions, differential diagnoses and treatments were also identified by PubMed searches when supplementary information was necessary. A comprehensive review of >800 articles formed the basis of discussion by three working groups (WGs). These groups focused on clinical diagnosis (WG1: A.L., Su.T., S.F.A., G.D.F., L.G., H.J., E.L.N., M.A.L., O.M., P.M., L.R., R.R., A.H.S., Se.T., P.W. and M.C.Z.), molecular diagnosis (WG2: G.P.d.N., M.B., D.M., T.E., F.M.E., K.F., P.H., M.-L.K., A.P., E.M.S. and C.S.) and clinical management (WG3: G.M., R.B., T.C., L.d.S., G.D., A.G.R., E.L.G.-L., N.H., O.H., P.K., N.K., B.L., R.M., G.A.M.-M., M.M., R.P., A.R., V.S. and A.U.).

Preparation for the consensus took >24 months, including two preparatory meetings. A preliminary document summarizing the questions addressed in the preparatory meetings was prepared by each WG and shared for review with all the experts before the final consensus meeting. At the final consensus meeting, propositions and recommendations were considered by participants and discussed in plenary sessions, enabling reformulation of the recommendations, if necessary. Where published data were unavailable or insufficient, experts’ clinical experiences and opinions were considered. Therefore, this Consensus Statement focuses on disorders for which we have sufficient published data and/or expertise, including PHP1A, PHP1B, PHP1C, PPHP, POH and acrodysostosis. All experts voted on the recommendations proposed by each working group using the following system: A. evidence or general agreement allows full agreement with the recommendation; B. evidence or general agreement is in favour of the recommendation; C. evidence or general agreement is weak for the recommendation; D. there is not enough evidence or general agreement to agree with the recommendation. If there was a majority of D, the recommendation was not accepted. Depending on the proportion of votes received by the option with the most votes, the strength of the recommendation was recorded as follows: + (26–49% of the votes), ++ (50–69% of the votes) and +++ ( ≥70% of the votes).

## Clinical diagnosis

PHP and related disorders vary considerably in clinical presentation and disease severity between affected individuals, even among patients carrying the same genetic alteration. The clinical symptoms (for instance, ossifications and brachydactyly) and abnormalities that can be detected in a laboratory (for instance, hypocalcaemia and raised levels of PTH) (Table 1) typically worsen during mid and late childhood and are usually unnoticed in very young children. A correct diagnosis can thus be elusive during infancy and in patients with atypical features^23^.

**Table 1:** Main clinical features of PHP and related disorders

Feature	PHP1A	PHP1B	PPHP	POH	ACRDYS1	ACRDYS2
Growth	• Growth velocity decreasing progressively • Adult short stature	• Macrosomia • Average adult stature	• SGA • Growth velocity decreasing progressively • Adult short stature	SGA	• SGA • Adult short stature	• SGA • Adult short stature
Obesity	Early onset	Early onset	Normal weight or lean	Normal weight or lean	Present	Present
Brachydactyly	70–80%	15–33%	<30%	Rare	97%	92%
Advanced bone age	70–80%	15–33%	Unknown	Unknown	100%	100%
Ectopic ossification	30–60%	0–40%	18–100%	100%	0%	0%
PTH resistance (progressive)	100%	100%	Rare and mild	Absent	100%	29%
TSH resistance	100%	30–100%	Rare and mild	Absent	~100%	16%
Neurological symptoms	• Neurocognitive impairment • Cerebral calcifications	Cerebral calcifications	Unknown	Unknown	Unknown	Neurocognitive impairment
Gonads	Gonadotropin resistance	Normal	Normal	Unknown	Case reports of anatomical dysfunction	Unknown

Pseudohypoparathyroidism (PHP) and related disorders affect many organs unequally. The clinical and biochemical features of the main diseases have been represented with their frequency when known. ACRDYS1, acrodysostosis due to mutation in *PRKAR1A*; ACRDYS2, acrodysostosis due to mutation in *PDE4D*; PHP1A, pseudohypoparathyroidism type 1A due to maternal loss of function mutation at the *GNAS* coding sequence; PHP1B, pseudohypoparathyroidism type 1B due to methylation defect at the *GNAS* coding sequence; POH, progressive osseous heteroplasia (due to paternal loss-of-function mutation at the *GNAS* coding sequence); PPHP, pseudopseudohypoparathyroidism (due to paternal loss of function mutation at the *GNAS* coding sequence); PTH, parathyroid hormone; SGA, small for gestational age.

### Clinical definitions

#### Albright hereditary osteodystrophy

The term AHO is used to indicate a constellation of physical features originally described by Albright^1^, including a round face, a stocky habitus with short stature, brachydactyly and ectopic ossification. Short bones are not present at birth and result from premature closure of the epiphyses, leading to a reduced period of growth. Although all bones tend to be short, shortening is most marked acrally (that is, in the hands and feet). Subsequently, developmental delay was added as an additional feature of AHO^24,25^. Obesity, particularly early-onset obesity, and macrocephaly relative to height might be part of AHO^26–28^.

#### Pseudohypoparathyroidism

The demonstration that levels of G_s_α, the α-subunit of the heterotrimeric G protein that couples heptahelical receptors to activation of adenylyl cyclase, were reduced in erythrocytes from some patients with PHP led to the first subclassification within PHP^29^.

PHP1A was initially defined as the association of resistance to multiple hormones, including PTH and TSH, features of AHO and decreased G_s_α activity using in vitro assays^29^. Evidence of TSH resistance is often present at birth and can lead to the misdiagnosis of congenital hypothyroidism^24,30^, whereas PTH resistance develops during childhood^31–34^. Similarly, brachydactyly develops progressively and usually becomes obvious before puberty^34,35^. Additional features have been described in patients with PHP1A. For instance, affected patients are born with a moderately reduced birth length that usually does not prompt investigations^24,30,36–38^. Obesity might develop in very early life and be recognized before any endocrine disturbances appear in early childhood^30,35,39^. The presence of extensive or progressive ossifications that extend deep into connective tissues is unusual but does not preclude the diagnosis of PHP1A^40,41^. Cognitive impairment has been associated with the diagnosis of PHP1A; when present, the severity of impairment is highly variable^24,42^. However, about 30% of patients with PHP1A present with normal cognitive development^24^.

PHP1C has been defined as the association of all the features of PHP1A but with documentation of normal G_s_α activity in in vitro complementation assays that do not assess the ability of receptors to activate the G_s_ heterotrimer^43^. As a result of the overlap with PHP1A and of the lack of assessment of G_s_α activity in most reports (and thus the lack of distinction between PHP1A and PHP1C), we have not specifically addressed PHP1C. This form is considered to be a variant of PHP1A in the text and the recommendations unless otherwise specified.

PHP1B was initially defined as isolated resistance to PTH, absence of AHO and normal levels of G_s_α activity. Subsequent analyses have demonstrated that some patients with PHP1B display additional features that overlap with PHP1A. As in PHP1A, PTH resistance might not be present at birth and develops only over time^44–50^. TSH resistance and patterns of excessive intrauterine growth or weight gain at birth or during early infancy and childhood have been described^44,51,52^. Some patients with PHP1B present with one or several features of AHO, the most frequent being brachydactyly^51,53^. Subcutaneous ossifications are very uncommon^51,53,54^. Owing to the overlap of some AHO features, distinguishing between PHP1A and PHP1B can be difficult in some patients; even more so as, although rarely investigated, mildly decreased G_s_α activity has also been described in some patients with PHP1B^55^.

PHP type 2 (PHP2) is characterized by an increase in levels of cAMP in response to exogenous PTH infusion but a deficient phosphaturic response^56^. The exact molecular cause of this disease variant is still unknown, but it has been suspected that PHP2 could either be an acquired defect secondary to vitamin D deficiency^57^ or be due to defective signalling downstream of G_s_α, as in patients with acrodysostosis due to *PRKAR1A* mutations^6^.

#### Pseudopseudohypoparathyroidism

PPHP is defined as AHO, with decreased G_s_α activity, in the absence of PTH resistance. Birthweight and length restriction are frequent^36^. Subcutaneous ossifications are also frequent (osteoma cutis and bony plaques)^58,59^ and highly suggestive of G_s_α deficiency. In some cases, mild resistance to PTH and TSH might occur^60^.

#### Progressive osseous heteroplasia

POH is defined by the presence of ectopic ossifications that are progressive and extend deep into connective tissue. At least one bony plate is present. Patients with POH usually have no other features of AHO and have normal responsiveness to PTH. The ossifications might lead to severe ankyloses of affected joints and focal growth retardation. Several clinical and genetic features can be suggestive of the diagnosis, such as a mutation involving exons 1–13 of the paternal *GNAS* allele, radiographic evidence of a reticular pattern of ossification, histological evidence of exclusive intramembraneous ossification or both intramembraneous and endochondral ossification, lateralization of the ossifications in a dermomyotomal pattern, being born small for gestational age (SGA), leanness and onset of ossifications before 1 year of age^10^. POH-like features have also been described in several patients in whom the *GNAS* mutations were located on the maternal allele^41^.

#### Acrodysostosis

Acrodysostosis is defined as the association of severe brachydactyly, facial dysostosis and nasal hypoplasia. Brachydactyly usually affects all phalanxes, metacarpals and metatarsals except for thumbs and halluces. On radiographs, epiphyses display a cone shape, bone age is advanced and abnormalities might be present at birth or soon thereafter. In some cases, the chondrodysplasia is indistinct from that of AHO^11–13,15,61^. Other symptoms might also be present in patients with acrodysostosis, such as cognitive impairment^15,62^, being born SGA and resistance to PTH and/or other hormones that signal through G_s_α^6,13,15,63,64^.

#### Effect of correct diagnosis

Confirmation of the diagnosis is important for patients and for parents of children with PHP and related disorders, as this guides appropriate management and prevents further, sometimes extensive, investigation of frequent presenting signs, such as growth failure, obesity and/or seizures, to identify the underlying cause. Conversely, exclusion of the diagnosis will prompt further investigation and consideration of alternative underlying conditions.

Establishing the correct diagnosis in patients with PHP, PPHP, POH or acrodysostosis allows an appropriate conversation to take place with patients and families, including anticipatory guidance, and informs decisions on biochemical screening and treatment of potential endocrine defects. Families can be directed to appropriate support groups, and genetic counselling can be offered. Identifying patients with these disorders is also important to allow further research into the underlying incidence, natural history and aetiology of the disease phenotypes.

***Recommendations***

1.1. The diagnosis of PHP and related disorders should be based on clinical and biochemical characteristics, which will vary depending on the age of the patient and, in some cases, on the family history (A+++).

1.2. In the context of PHP and related disorders, the diagnosis of AHO should be based on the presence of the following clinical features:

- Major criterion: brachydactyly type E (premature fusion of the epiphyses)
- Major criterion: short stature by adulthood relative to the height of the unaffected parent
- Additional criterion: stocky build
- Additional criterion: round face in comparison with siblings and degree of obesity, if present
- Additional criterion: ectopic (and often subcutaneous) ossifications

All features might not be present and might evolve throughout the observation period. Obesity, SGA, dental manifestations and cognitive impairment are present only in a subgroup of patients; these features are not required for the diagnosis of AHO (A++).

1.3. Clinical and biochemical major criteria for PHP and related disorders are as follows (A+++):

- PTH resistance
- And/or subcutaneous ossifications that can include deeper ossifications
- And/or early-onset (before 2 years of age) obesity associated with TSH resistance or with one of the above
- And/or AHO alone
- With or without a family history

1.4. The following features support the diagnosis of PHP and related disorders (A+++):

- Endocrine: elevated levels of TSH, unexplained congenital hypothyroidism (mildly elevated levels of TSH), hypogonadism, hypercalcitoninaemia and/or GH deficiency
- Neurological: cognitive impairment, hearing impairment, spinal stenosis, Chiari malformation type 1, syringomyelia, carpal tunnel syndrome and/or craniosynostosis
- Mineralization defects: enamel hypoplasia, delayed tooth eruption or tooth ankylosis, oligodontia or hypodontia, advanced skeletal maturation, cataract and/or central nervous system (CNS) calcifications
- Others: sleep apnoea, ear infection, asthma, early-onset obesity, SGA and/or cryptorchidism

1.5. PHP and related disorders are primarily clinical diagnoses. Identification of the molecular cause should be performed to confirm the clinical diagnosis and allow the characterization of the subtype of the disease (A++).

1.6. Testing for genetic or epigenetic causes should be based on the clinical characteristics, local access to genetic testing and the most likely identified causes of the disease at the time of analysis according to the algorithm (see Molecular diagnosis section) (A++).

The experts have highlighted that administration of exogenous PTH (modified Ellsworth–Howard test) is not necessary but might be helpful in research settings. Assessment of G_s_α bioactivity is usually not required for the clinical diagnosis of PHP and related disorders.

### Main clinical components

In the majority of patients with PHP, the most important clinical manifestation is symptoms of hypocalcaemia due to PTH resistance (45–80%)^65^. Periods of rapid growth and the associated increased calcium requirement, or nutritional calcium or vitamin D deficiency, might trigger or intensify symptoms^34^.

#### Resistance to PTH

In patients with PHP1A, resistance to PTH is usually absent at birth and evolves over life (from 0.2 years to 22 years)^18,31,34^, while the clinical manifestations typically occur later. These data suggest that PTH resistance begins in early childhood, and the resultant changes in serum levels of calcium and phosphorus develop gradually, at some point during adulthood^31,32,66–68^. The first biochemical abnormalities to become apparent are elevated serum levels of PTH and elevated serum levels of phosphorus, followed by hypocalcaemia. When hypocalcaemia is present, urine levels of calcium are low, whereas calcitriol levels might be either low or normal^18^. An interval of up to 4.5 years usually occurs between the start of the elevation in levels of PTH and phosphorus and onset of hypocalcaemia^67^.

Patients with PHP1B might show variable degrees of PTH-resistant hypocalcaemia or normocalcaemia, despite identical epigenetic changes involving transcription start site (TSS)–differentially methylated region (DMR) at exon A/B of GNAS (*GNAS A/B*:TSS-DMR)^47,66,69,70^. Long-standing secondary hyperparathyroidism with chronic hypocalcaemia and calcitriol deficiency has been associated with tertiary hyperparathyroidism in these patients^71^. Chronically elevated levels of PTH might also lead to bone resorption and demineralization that resemble the bone changes seen in rickets in children with irregular and widened metaphyses, Madelung-like deformity or primary hyperparathyroidism, including brown tumours (a bone lesion that can occur when osteoclast activity due to hyperparathyroidism is excessive)^72^.

Resistance to PTH is also present in patients with mutations in *PRKAR1A*^5,6,15,20,63,64^; however, hypocalcaemia has not been documented in these patients yet. Patients with *PDE4D* mutations usually display normal levels of PTH, except in the context of calcifediol deficiency^5,13–15,20,62,63^.

***Recommendations***

1.7. The definition of PTH resistance is as follows:

- The association of hypocalcaemia, hyperphosphataemia and elevated serum levels of PTH in the absence of vitamin D deficiency and when magnesium levels and renal function are normal.
- PTH resistance in the context of PHP and related disorders should be suspected when PTH is at, or above, the upper limit of normal, in the presence of normal calcifediol levels and elevated serum levels of phosphorus, even in the absence of overt hypocalcaemia.

PTH resistance and consequent changes in serum levels of calcium, phosphorus and PTH can be variable, and repeated testing might be required (A+++).

#### Ectopic ossification

Disorders caused by molecular alterations of the *GNAS* gene or locus, such as PHP1A, PHP1C, PPHP and POH, can feature ectopic ossification. Ectopic ossification is not calcification and is unrelated to serum levels of calcium and phosphorus. The ectopic ossifications are a manifestation of G_s_α deficiency in mesenchymal stem cells, with de novo formation of extraskeletal osteoblasts that form islands of ectopic bone in the dermis and the subcutaneous fat as a result of the differentiation of adipose-derived progenitor mesenchymal stem cells that lead to predominantly web-like intramembranous ossifications^73,74^. Unlike fibrodysplasia ossificans progressiva (FOP), where injury, viral infections and immunizations can lead to ossifications, no formal evidence exists to suggest that the same events lead to ectopic bone formation in *GNAS*-based conditions. Ectopic ossifications are found in 100%, 80–100%, 30–60% and very uncommonly in patients with POH, PPHP or AHO, PHP1A and PHP1B, respectively, and have never been reported in patients with acrodysostosis^13,61,75–78^.

Osteoma cutis (a form of ectopic ossification) is usually present at birth or develops very early in life, more rarely during childhood and adulthood, as a single large plaque in the skin, as an isolated dermal nodule or nodules or as multiple papules on the face^79^. Osteoma cutis is associated with normal serum levels of calcium and phosphorus throughout life, which suggests that the mutation is on the paternal *GNAS* allele^41,58,59,80–82^. Ossifications that develop in patients with POH are progressive and extend into the muscles, tendons and ligaments^10,41,83^. The ossifications in patients with POH can be limited to or be much more prominent on one side of the body, which suggests that the mechanism of the disease encompasses a second mutational mosaic hit or variations in *GNAS* imprinting or G_s_α expression^84^.

***Recommendations***

1.8.  Ectopic ossifications should be considered as a specific sign of *GNAS* mutations (specifically when observed at birth or in early childhood) (A++).

1.9.  The description of the ossifications, their location and their extension in connective tissues should be considered to establish the diagnosis of POH versus osteoma cutis, PHP1A, PHP1C, PPHP or AHO, where the latter conditions have ectopic bone that remains superficial (B++).

1.10. The diagnosis of osteoma cutis, nodules of subcutaneous ossifications or deep heterotopic ossifications should trigger a clinical and biochemical work-up to search for signs of AHO, PTH and TSH resistance or FOP, especially if the first digit of both feet is abnormal. If the diagnosis is obvious or very likely, a biopsy sample is not needed and is contraindicated in case of suspicion of FOP (A+++).

#### Brachydactyly

In PHP and related disorders, brachydactyly can be classified as type E, which is defined as variable shortening of the metacarpals with, usually, normal length of phalanges, occasionally accompanied by relatively shortened metatarsals. In PHP and related disorders, the fifth, fourth and third metacarpal and the first and fourth distal phalanges are the most affected bones of the hand^85^; metatarsals are often shortened as well. Brachydactyly develops over time and might not be evident in early life, except in patients with acrodysostosis^6,35,86^. The frequency and severity of brachydactyly vary among the different disorders: 70–80% in PHP1A^51^, 15–33% in PHP1B^46,51,70,87–93^ and all patients with acrodysostosis^5,6,14,15,20,62–64,94–96^ (Table 1). However, brachydactyly is not specific to PHP and related disorders and can be found in patients with, for example, tricho–rhino–phalangeal syndrome, brachydactyly mental retardation syndrome or Turner syndrome (Table 2).

**Table 2:** Differential diagnoses of PHP and related disorders based on the main clinical presentation

Leading symptom	Differential diagnosis	Associated signs or comments
Hypocalcaemia with elevated PTH	Vitamin D deficiency or resistance^57,130^	• Improvement upon vitamin D therapy • Rickets and alopecia also seen
	Rickets^268^	Enlargement of the metaphyses, leg bowing and elevated ALP
	Hypoparathyroidism due to a mutation in the *PTH* gene^269^	Use different assays to confirm the elevated PTH
Brachydactyly	Tricho–rhino–phalangeal syndrome due to *TRPS1* mutations^259^	• Dysmorphism: slowly growing and sparse scalp hair, laterally sparse eyebrows, bulbous tip of the nose, long flat philtrum, thin upper vermillion border and protruding ears • Hip dysplasia, small feet and a short hallux, exostosis and ivory epiphyses
	Isolated brachydactyly type E due to *HOXD13* mutations^270^	• Syndactyly, long distal phalanges and shortening of the distal phalanx of the thumb • Hypoplasia or aplasia, lateral phalangeal duplication and/or clinodactyly
	Brachydactyly mental retardation syndrome due to 2q37 microdeletions^271^	Obesity, short stature, brachydactyly and psychomotor and cognitive alterations
	Turner syndrome due to partial or complete loss of one X chromosome^272^	Short stature, low birthweight, gonadal failure and variable neurocognitive defects; brachydactyly and Madelung deformity
	Brachydactyly type E with short stature due to *PTHLH* mutations^75,273^	Short stature of variable severity and impaired breast development; oligodontia, delayed tooth eruption and dental malposition; pseudoepiphyses and brachydactyly
Ossifications (subcutaneous)	Acne vulgaris^80^	Superficial nodules but no ossification at pathology
	Cutaneous tumours, primarily pilomatricomas, chondroid syringomas and basal cell carcinomas, and pilar cysts and nevi^80^; secondary or traumatic osteoma cutis and miliary osteoma	• No ossification at pathology • For secondary osteoma cutis: history of trauma and burn
	Inflammatory conditions such as scars, chronic venous stasis, morphea, scleroderma, dermatomyositis and myositis ossificans progressiva^80^	No ossification at pathology
Ossifications (progressive)	FOP due to a recurrent activating missense mutation of *ACVR1* (ref.^10^)	Progressive ossification of skeletal muscle, tendons, fascia and ligaments; upper back and neck are the first parts of the skeleton to be affected; trauma alters the natural progression of the disease; congenital malformation of the great toes
	Tumoural calcinosis due to *FGF23* or *GALNT3* mutations^274^	Deposition of calcium within the skin and/or muscles and hyperphosphataemia
Early-onset obesity	Beckwith–Wiedemann syndrome^104,210^	Hemihypertrophy and macroglossia
	Genetic, cytogenetic or syndromic anomalies associated with early-onset obesity, including Prader–Willi syndrome and monogenic obesity (mutations in *POMC, MC4R*, leptin and the leptin receptor)^46,275^	Progression of obesity through childhood; possible associated features, such as red hair and hypoadrenalism
Early-onset hypothyroidism	Congenital hypothyroidism of any cause^46,47^	Small thyroid and TSH moderately elevated; no other associated features
	TSH resistance due to mutations in the TSH receptor^276^	Small thyroid and TSH moderately elevated; no other associated features
Hypertension	Autosomal dominant hypertension and brachydactyly type E syndrome^239^	Short stature

The list of differential diagnoses is not exhaustive but mentions the main diseases that overlap with pseudohypoparathyroidism (PHP) and related disorders. ALP, alkaline phosphatase; FOP, fibrodysplasia ossificans progressive; PTH, parathyroid hormone.

***Recommendations***

1.11. When brachydactyly type E is identified, other disorders associated with bone dysplasia should be excluded, for example, Turner syndrome, tricho–rhino–phalangeal syndrome, isolated brachydactyly type E and brachydactyly mental retardation syndrome (A+++).

1.12. If not present in infancy, brachydactyly type E should be searched for (clinically and radiologically) from early childhood onwards in all patients with PHP and related disorders (A+++).

### TSH resistance

Patients with PHP1A frequently (if not always^97^) present with raised serum levels of TSH and thyroid hormone levels that are normal or slightly reduced. Some patients present with overt clinical hypothyroidism. Elevated levels of TSH due to TSH resistance might be present at birth and detected on neonatal screening^24,30,31,60,67,98–100^. In PHP1B, TSH levels are at the high end of normal or mildly elevated in 30–100% of patients^47,87,89,92,101–105^. TSH resistance is present in patients with acrodysostosis owing to *PRKAR1A* mutations but not in those with *PDE4D* mutations^13,15^. Thyroid autoantibodies are usually absent in patients with PHP1A, PHP1B or acrodysostosis who have elevated levels of TSH. Nevertheless, given the high prevalence of autoimmune thyroid disease, the presence of thyroid autoantibodies does not mean that the patient might not also have TSH resistance.

### Additional frequent clinical features

Many aspects of PHP and related disorders do not lead to measurable hormonal changes and are not yet recognized as being part of the spectrum of PHP-related disorders (Table 1).

SGA has been described in patients with a paternal *GNAS* mutation (that is, patients with PPHP or POH)^106^. Similarly, most patients with acrodysostosis and a mutation in *PRKAR1A* or *PDE4D* are born SGA^5,13,15^. Most patients born SGA who have PHP or related disorders remain short during their adult life (with a mean final height in PPHP, AHO, PHP1A and acrodysostosis <−2 s.d.)^13,15,23,86^. By contrast, patients with methylation changes at the *GNAS* locus display moderately increased birthweight and birth length (A.L., H.J., A.H.S., G.M. and A.R., unpublished observations). Their final adult height is within the normal range^107^.

Although they are unspecific features, overweight and obesity are associated with specific PHP-related disorders, such as PHP1A, PHP1C and acrodysostosis^5,6,15,23,28,39,62,64^. Patients with PHP1A and PHP1B can develop early-onset obesity, usually in the first 2 years of life^28,44,49,108–111^. Noticeably, obesity in adulthood is less severe and less common than in childhood^28^. Patients with PHP1A might also experience reduced insulin sensitivity^112,113^.

Cognitive impairment is commonly reported in patients with PHP or related disorders; however, its exact prevalence is not known, as the results of objective standard tests are rarely reported (40–70% of patients with PHP1A, 0–10% of patients with PPHP or POH, rare in patients with PHP1B and of variable prevalence in patients with acrodysostosis)^1,18,23,24,26,42^.

Elevated levels of calcitonin are found in a large subset of patients with PHP1A and PHP1B, after exclusion of medullary thyroid carcinoma. Elevated levels of calcitonin might be considered as a marker of hormone resistance in a patient with a PHP or related disorder^114,115^

Resistance to gonadotropins seems to be less severe than resistance to other hormones, such as PTH and TSH; laboratory abnormalities indicating elevated levels of luteinizing hormone (LH) or follicle-stimulating hormone (FSH) have been reported by some groups^116–121^, whereas others could not confirm these findings^122–127^. These findings have led to the hypothesis that patients with PHP1A display only partial resistance to gonadotropins^120^.

Although nonspecific symptoms such as early-onset obesity, short stature and/or hypothyroidism might be present in early infancy or childhood, the diagnosis is often delayed or not recognized until early puberty for patients with PHP1A and acrodysostosis or until adolescence or adulthood for patients with PHP1B, unless the family history is positive. The presence of ectopic ossifications (which are disease-specific) might trigger an earlier diagnosis of POH or osteoma cutis.

### Differential diagnoses

The differential diagnosis of patients with obesity, early-onset (even congenital) hypothyroidism, short stature and/or brachydactyly is very broad and includes many endocrine and syndromic diseases (Table 2). Some features, however, should prompt the clinician to consider diagnoses other than PHP or related disorders. These features include additional bone anomalies, such as syndactylies, exostoses or Madelung deformity^128^, and/or facial dysmorphism or other features suggestive of Turner syndrome or tricho–rhino–phalangeal syndrome^72,129^. The main differential diagnosis of ectopic bone formation is FOP. Severe vitamin D deficiency or hypomagnesaemia might mimic PTH resistance^130,131^. A correct diagnosis can have extremely important implications for management, as it enables early screening and treatment of endocrine complications such as PTH and TSH resistance, prevention and management of obesity and short stature, management of ossifications and accurate genetic and prenatal counselling.

### Evolution of PHP classification

The first classification system distinguished PHP variants as PHP1A, PHP1B, PHP1C and PPHP^1,2,27,29,69,132,133^. The subtype assignment is based on the presence or absence of AHO together with characterization of hormone resistance and determination of G_s_α protein activity using in vitro assays^132,133^. In addition, this classification excludes phenocopies of PHP, such as acrodysostosis and POH. Since the diseases were molecularly characterized, it has become evident that clinical phenotypes often fail to differentiate between PHP subtypes^43,103,134,135^. The overlap between the diseases (Table 1 and Supplementary Table 1) renders the diagnosis complex and the classification inadaptable and obsolete. In addition, the tools to investigate patients have evolved, measurement of G_s_α activity in cell membranes is not readily available and molecular genetic diagnosis has become the gold standard by which PHP variants are distinguished^17^.

***Recommendations***

1.13. Consider genetic diagnosis in patients who present with one or more major criteria suggestive of PHP and related disorders (A+++).

1.14. The classification of PHP and related disorders should be amended to include the following (A++):

- A common pathophysiological framework
- A molecular genetic classification

## Molecular diagnosis

A positive molecular test provides important confirmation of the clinical diagnosis and allows the categorization of a patient into a specific subtype of PHP, which can guide management.

Annotation of each variant (including the interpretation of the variant according to international guidelines^136^) and its association with the clinical findings within the curated Leiden Open Variation Database (LOVD) could improve our knowledge of the correlation between molecular defects and phenotype.

### Molecular confirmation

#### GNAS locus

As previously mentioned, the main subtypes of PHP are caused by de novo or autosomal dominantly inherited inactivating genetic pathogenic variants or epigenetic alterations (sporadic or genetic-based) within or upstream of the *GNAS* locus. This region gives rise to multiple non-coding and coding transcripts, including those encoding G_s_α (Supplementary Fig. 1a). *GNAS*, the gene that encodes G_s_α, is an imprinted gene. It shows biallelic expression in most studied tissues, whereas primarily maternal expression is observed in some tissues (thyroid, renal proximal tubule, pituitary and ovary)^52,137–140^. This tissue-specific monoallelic expression of G_s_α explains most of the clinical outcomes that depend on the parental origin of the *GNAS* mutation. For instance, because the paternal G_s_α allele is mostly silenced in the renal proximal tubule, *GNAS* mutations on this allele do not impair G_s_α activity. By contrast, if a *GNAS* mutation is inherited maternally or if it occurs de novo on the maternal allele, then G_s_α levels and/or activity are drastically reduced, leading to PTH resistance in the renal proximal tubule. The molecular and genetic mechanisms underlying *GNAS*-related disorders are reviewed further in multiple other articles^33,141,142^.

In brief, PHP1A is caused by inactivating variants on the maternal allele of the *GNAS* gene within exons 1–13 (referring sequences NG_016194.1/NM_001077488.1 and LRG_1051), including both point mutations and rare gene rearrangements^16,23,27,41,45,86,88,97,112,122,143–148^ (Supplementary Fig. 1b). Mutations can be either maternally inherited or de novo, with both types of mutation having similar incidences^17^. When mutations affect the paternal allele, they mainly cause PPHP but can also be responsible for osteoma cutis or POH^23,41,83,86,122,145,147,149^. Point mutations can be easily detected by sequencing (either Sanger sequencing or next-generation sequencing (NGS); particular attention should be given to exon 1 in the latter method, as this exon is particularly CG-rich and coverage might be incomplete^150^), whereas genomic rearrangements can be analysed by quantitative methods, such as multiplex ligation-dependent probe amplification (MLPA) or comparative genomic hybridization arrays (aCGH)^144^.

By contrast, patients with PHP1B show abnormal patterns of methylation in the DMRs associated with the *GNAS* complex locus^45,47,86–88,101,143,151–154^. A methylation defect can be classified as partial or complete and can affect one or multiple DMRs within 20q13 (ref.^155^). *GNAS* shows differential methylation at four distinct DMRs: one paternally methylated-DMR (*GNAS-NESP*:TSS-DMR) and three maternally methylated-DMRs (*GNAS-AS1*:TSS-DMR, *GNAS-XL*:Ex1-DMR and *GNAS A/B*:TSS-DMR, according to the current nomenclature^156^). Loss of methylation at *GNAS A/B*:TSS-DMR is detected in all patients with PHP1B^45,47,86–88,101,143,151–154^.

Of the PHP1B cases, 15–20% are familial, with an autosomal dominant mode of inheritance (AD-PHP1B) through the maternal lineage^17^. In this familial form, the methylation defect is usually limited to loss of methylation at *GNAS A/B*:TSS-DMR, secondary to a 3 kb microdeletion on the maternal allele of *cis-*acting control elements within *STX16* (ref.^157^). Other maternally inherited deletions and duplications have also been identified in some rare familial cases affecting either an isolated *GNAS A/B*:TSS-DMR^66,158–160^ or all four DMRs^50,159,161–163^ (Supplementary Fig. 1c).

In nearly all sporadic cases of PHP1B, two or more DMRs, in addition to *GNAS A/B*:TSS-DMR, are also affected, for which no underlying genetic mechanism has been identified^154^ (Supplementary Fig. 1d). In around 8–10%^17,102^ of these sporadic cases, the methylation defects are caused by paternal uniparental isodisomy of the chromosomal region comprising *GNAS* (UPD(20q)pat)^164–168^ (Supplementary Fig. 1e).

To date, very few cohorts of patients with PHP1B have been extensively screened for multilocus imprinting disturbance (MLID). The incidence of MLID associated with PHP1B ranges from absent^169^ to 38%^92^, with most studies quoting a frequency in between these values. This variation can be partially explained by the small number of patients investigated in each study, the low sensitivity of molecular techniques or the total number of imprinted loci assessed. Consistent with most studies of MLID, patients with PHP1B-MLID do not show evidence of phenotype differences, and robust methylation changes are restricted to those with a sporadic cause^89,92,104,170,171^. Despite the important advances made with high-density methylation array screening, bioinformatic standardization is required to ensure accurate comparisons between cohorts and accurate description of methylation disturbance in individual patients.

*GNAS* methylation defects can be detected through the use of several methods. Methylation-sensitive MLPA (MS-MLPA) enables interrogation of multiple regions in a single reaction. A kit from MRC-Holland is available (MS-MLPA ME031 GNAS) to determine the level of methylation at multiple *GNAS* sites and to screen for *STX16* and *NESP/AS* deletions and deletions encompassing *GNAS*^172^. Alternative techniques used for the detection of methylation defects, but that cannot discriminate epigenetic abnormalities as a result of *GNAS* deletions, include combined bisulfite restriction analysis (COBRA), pyrosequencing, methylation-sensitive single nucleotide primer extension (MS-SNuPE) and EpiTYPER^155,157,169^.

When testing for disomy, the identification of the two (identical) replica copies of a single homologue of a paternal 20q chromosome can be analysed either by microsatellite or short tandem repeat (STR) typing (analysis of the trio, that is, parents and the index case, might be essential for definitive conclusions) or by performing a single-nucleotide polymorphism (SNP) array^164,165,167^.

On the basis of the different molecular alterations, molecular testing must be able to robustly and accurately detect point mutations, genetic or genomic rearrangements and methylation defects.

#### PRKAR1A and PDE4D

Only heterozygous point mutations in *PRKAR1A* (referring sequence NC_000017.11/NM_002734.4) or *PDE4D* (referring sequence NG_027957.1/NM_001165899) have been associated with acrodysostosis^5,6,13–15,20,62–64^.

Other genetic or genomic alterations have been identified in these genes, but they are not associated with PHP and related disorders. For example, heterozygous genomic rearrangements (deletions and duplica-tions) at 5q12.1 encompassing *PDE4D* are associated with a novel intellectual disability syndrome (OMIM #615668) without acrodysostosis and with a specific phenotype that diverges from that of patients with point mutations in *PDE4D*^14^. Furthermore, mutations and deletions in 17q24.2-q24.3 encompassing *PRKAR1A* have been associated with Carney complex^173^, which results from activation of protein kinase A signalling^174^.

***Recommendations***

**Fig. 2:**
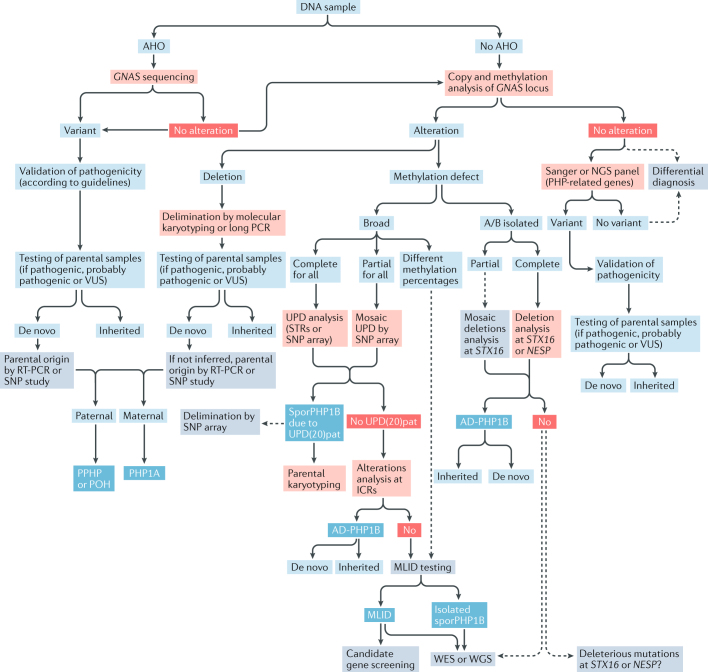
Molecular algorithm for the confirmation of diagnosis of PHP and related disorders. If patients present with Albright hereditary osteodystrophy (AHO), genetic alterations at *GNAS* should be studied, including point mutations (sequencing) and genomic rearrangements (such as multiplex ligation-dependent probe amplification (MLPA) and comparative genomic hybridization arrays (aCGH)). Once the variant is found, its pathogenicity should be confirmed according to guidelines^136^, and, when possible, the parental origin should be determined. In the absence of AHO, epigenetic alterations should be analysed first. According to the results obtained for the methylation status, further tests are needed to reach the final diagnosis: if the methylation defect is restricted to transcription start site (TSS)–differentially methylated region (DMR) at exon A/B of GNAS (*GNAS A/B*:TSS-DMR), *STX16* deletions should be screened for, and, if present, the diagnosis of autosomal dominant-pseudohypoparathyroidism type 1B (AD-PHP1B) is confirmed; if the methylation is modified at the four DMRs, paternal uniparental disomy of chromosome 20 (UPD(20q)pat) should be screened for; in absence of UPD(20q)pat, deletions at *NESP* should be screened for; if no genetic cause is identified as the cause of the methylation defect, the sporadic form of the disease (sporPHP1B) is suspected. After exclusion of the *GNAS* locus as the cause of the phenotype, and in patients with AHO, pseudohypoparathyroidism (PHP)-related genes (that is, at least *PDE4D* and *PRKAR1A*) should be sequenced. Squares in light red indicate the technology; blue, the final molecular confirmation; red, no molecular alteration; and grey, future or research steps are suggested. ICRs, imprinting control regions; MLID, multilocus imprinting disturbance; NGS, next-generation sequencing; PHP1A, pseudohypoparathyroidism type 1A; PHP1B, pseudohypoparathyroidism type 1B; POH, progressive osseous heteroplasia; PPHP, pseudopseudohypoparathyroidism; RT-PCR, reverse-transcription PCR; SNP, single-nucleotide polymorphism; STRs, short tandem repeats (microsatellites); UPD, uniparental disomy; VUS, variant of unknown significance; WES, whole-exome sequencing; WGS, whole-genome sequencing.

2.1. Molecular diagnosis of individuals with a suspected diagnosis of PHP must include DNA sequence, methylation and copy number variant (CNV) analyses at the *GNAS* locus (Fig. 2) (A++).

2.2. When the clinical pattern is highly suggestive of alteration of a specific gene, Sanger sequencing of that gene is proposed (A++).

2.3. When the clinical presentation is not suggestive of a specific gene, a targeted gene panel encompassing genes that encode proteins involved in the PTH–parathyroid hormone-related protein (PTHrP) signalling pathway might be performed. If gene panel sequencing is used, it should fulfil the recommended guidelines^175^ for design and reporting (A++).

2.4. If a sequence variant is identified in any gene associated with PHP and related disorders, its pathogenicity should be assessed according to the established standards and guidelines^136^ (A+++).

2.5. The parental origin of the variant could have important clinical implications; thus, parental testing is indicated when a genetic alteration is detected (A++).

2.6. If the variant is de novo, the allelic origin might have to be determined (B+++).

2.7. In certain cases, such as UPD(20q)pat or gross deletions, additional genetic studies might be advisable (A++).

2.8. Technical limitations must be taken into consideration when reporting negative results and included in the genetic report. For instance, mosaicism can be considered if a negative result is observed in a patient with high clinical suspicion through analysis of tissues other than blood (for example, saliva or a buccal swab) or the use of alternative techniques, such as deep sequencing (A++).

2.9. For each de novo pathogenic variant, or variants of unknown significance, parental germline mosaicism should be considered (A++).

2.10. After exclusion of alterations in genes associated with PHP and related disorders, the patient should be referred to an expert centre (A++).

### Genetic counselling

Despite the clinical and molecular overlap between PHP and related disorders (Table 1 and Supplementary Table 1), once the molecular defect is identified, genetic counselling is available for patients and families in most expert centres.

No correlation exists between the severity of the parental phenotype and that of the affected offspring, as interfamilial and intrafamilial variability has been observed for the same mutation, and the imprinting effect at the *GNAS* gene also affects clinical outcomes^16,64^.

When dealing with *GNAS* gene defects, the parental origin of the mutation and clinical heterogeneity make genetic counselling a challenging task, mainly owing to four factors. First, paternal inheritance (or a mutation in the paternal allele) can lead to either a mild expression of PPHP or a severe expression of POH. Why paternal inheritance of a *GNAS* mutation should result in PPHP in some families and POH in others remains unclear^10,74,84^. Second, when the mutation involves the maternal allele, it will lead to PHP1A or PHP1C. Third, patients have a 50% chance of transmitting the molecular defect, and depending on their sex, the descendant will develop PPHP or POH (when the patient is male) or PHP1A or PHP1C (when the patient is female). Fourth, there is no clear correlation between the type or location of *GNAS* mutations and the disease onset, severity of endocrine resistance, neurocognitive phenotype or the number of AHO features.

When methylation alterations at the *GNAS* locus are present, the recurrence risk depends on the underlying genetic defect, if present, and the parental origin. Paternally inherited *STX16* or *NESP/AS* deletions are not associated with methylation defects, so that the descendants of a male carrying any of these deletions have a 50% chance of inheriting the genetic defect; however, if they inherit the genetic defect, their *GNAS* methylation status will be normal, and they will not develop PHP1B^157^. If a female carries the *STX16* or *NESP/AS* deletion, each of her descendants has a 50% risk of inheriting the genetic defect, and if the descendant inherits the defect, he or she will present with a methylation defect affecting only *GNAS A/B*:TSS-DMR or the complete *GNAS* locus (depending on the inherited deletion)^18^. In both cases, the descendant will develop PHP1B^45,47,86–88,101,143,151–154^. Although translocations of chromosome 20 resulting in UPD(20)pat are very rare, parental karyotyping is recommended to establish the risk of recurrence. If no genetic alteration can be identified that is associated with the methylation defect, the recurrence and transmission risks are expected to be similar to that of the general population.

Pathogenic variants at *PRKAR1A* and *PDE4D* mostly occur de novo^5,6,13–15,20,62–64^. According to the pattern of autosomal dominant inheritance, patients have a 50% of chance of passing on the molecular defect and the disease to their descendants, independently of the sex of either the progenitor or the descendant.

The identification of a pathogenic variant in an index case enables a correct diagnosis and the possibility of predictive genetic testing in relatives, which can be excluded from further follow-up if the result is negative^176^. In parents of an index case, it is essential to exclude mosaicism^116,177,178^ or to indicate this possibility at the counselling session.

***Recommendations***

2.11. Genetic counselling should be adapted to the different molecular findings for the *GNAS* locus (B++) and the *PRKAR1A* and *PDE4D* genes (A++) according to Table 3.

**Table 3:** Guidance for genetic counselling in PHP and related disorders according to each molecular defect

Locus	Molecular diagnosis	Molecular defect	Affected allele	Cases (*n* or %)	Theoretical recurrence risk (when inherited)	Clinical predictive outcome	Further tests
*GNAS*^a^	Genetic defect	Heterozygous loss-of-function mutation	Mat	35.9%	50%	Affected pt PHP1A	Maternal testing for carrier status
			Pat	5.9%	50%	Affected pt PPHP and/or POH	Paternal testing for carrier status
		Partial or total locus deletion or inversion	Mat	1.8%	50%	Affected pt PHP1A	Maternal testing for carrier status
			Pat	0.2%	50%	Affected pt PPHP and/or POH	Paternal testing for carrier status
	Sporadic methylation defect	Broad LOI (all *GNAS* DMRs)	Mat	38%	ND	Affected pt sporPHP1B	Research
		Isolated LOM *GNAS A/B*:TSS-DMR	Pat	2%	ND	Affected pt sporPHP1B	Research
		Uniparental disomy (UPD(20q)pat)	–	2.7%	Low; high when a translocation is present	Affected pt sporPHP1B	Chromosome analysis of proband and father
	Inherited methylation defect	Isolated LOM *GNAS A/B*:TSS-DMR + *STX16* ICR deletion	Mat	13.5%	50%	Affected pt AD-PHP1B	Maternal testing for carrier status
		*STX16* ICR deletion	Pat	ND	50%	Unaffected carrier	Not required
		Broad LOI + *NESP/AS* ICR deletion	Mat	ND	50%	Affected pt AD-PHP1B	Maternal testing for carrier status
		*NESP/AS* ICR deletion	Pat	ND	50%	Unaffected carrier	Not required
*PRKAR1A*^b^	Genetic defect	Heterozygous mutation	Not relevant	79	50%	Affected pt	Parental testing for carrier status
*PDE4D*^b^	Genetic defect	Heterozygous mutation	Not relevant	43	50%	Affected pt	Parental testing for carrier status

AD-PHP1B, autosomal dominant pseudohypoparathyroidism type 1B; DMR, differentially methylated region; ICR, imprinting control region; LOI, loss of imprinting; LOM, loss of methylation; Mat, maternal; ND, not determined; Pat, paternal; PHP, pseudohypothyroidism; PHP1A, pseudohypoparathyroidism type 1A; POH, progressive osseous heteroplasia; PPHP, pseudopseudohypoparathyroidism; pt, patient; sporPHP1B, sporadic pseudohypoparathyroidism type 1B. ^a^Percentages obtained from Elli et al.^17^ ^b^Numbers extracted from the corresponding Leiden Open Variation Database (LOVD) web referred to total carriers with public variants (it varies with time).

### Prenatal testing

There are very few cases with antenatal diagnosis of PHP or PPHP, as their clinical manifestations (including growth retardation, subcutaneous ossifications and TSH resistance) are usually not specific within this period. The bone dysplasia observed in patients with acrodysostosis might display a prenatal onset, mostly when caused by *PRKAR1A* mutations^6^.

***Recommendations***

2.12. Even if all the molecular defects associated with PHP and related disorders were known and technically available for testing in preimplantation or prenatal diagnosis, ethical and legal concerns regarding the pertinence of performing these tests should be taken into consideration, with appropriate genetic counselling (A++).

2.13. If prenatal testing is considered, methylation analysis is not recommended (A++).

## Management

To date, no prospective clinical trials have been conducted in patients with PHP and related disorders that focus on the management and outcomes of treatment in these disorders. Large cohorts of molecularly diagnosed patients are needed for recruitment into clinical studies, which should also include long-term follow-up. As a result of the rarity of the disease, cohorts and research forces are scattered; there is a need to coordinate and implement multicentre international clinical trials.

A multidisciplinary follow-up and early, specific interventions are necessary for efficient therapeutic management of these patients. Table 4, which is based on the literature analysis and authors’ expertise, summarizes the main interventions that should take place during the follow-up of patients with PHP and related disorders and gives a suggested frequency for the interventions.

**Table 4:** Summary of the main interventions during the follow-up of patients with PHP and related disorders

Action points	Infancy (newborn to 2 years)	Early childhood (2–6 years)	Late childhood to adolescence	Adulthood
*Anticipatory guidance*				
Family support	✓	✓	✓	NA
Genetic counselling	At diagnosis	At diagnosis	At diagnosis	At diagnosis
*Medical evaluation*				
Linear growth	✓	✓	✓	NA
Weight gain and BMI	✓	✓	✓	✓
Descended testis	✓	✓	If not checked before	If not checked before
Blood pressure	NA	✓^a^	✓	✓
Development and/or cognition	✓	✓	S	S
Psychosocial evaluation	NA	✓	S	S
Ectopic ossifications	✓	✓	✓	S
Orthodontic and/or dental	NA	✓	✓	S
Bone age radiography	NA	✓ (in case of growth deceleration)	✓ (in case of growth deceleration)	NA
Calcium–phosphorus metabolism	✓	✓	✓	✓
Age-appropriate renal imaging	✓^b^	✓^b^	✓	✓^b^
Thyroid	✓	✓	✓	✓
Puberty	NA	NA	✓ (biochemistry in case of retardation)	NA
GH secretion	NA	✓	✓	S
Glucose and lipid metabolism	NA	✓	✓	✓
Fertility	NA	NA	S	S

GH, growth hormone; NA, not applicable; PHP, pseudohypoparathyroidism; S, subjective (by history and physical examination); ✓, to be performed at diagnosis and annually thereafter. ^a^At least once per year, with an appropriate sized cuff. ^b^Annually in case of increased excretion of urinary calcium or nephrocalcinosis.

***Recommendation***

3.1. The diagnosis and management of patients with PHP and related disorders should be performed by a multidisciplinary team of specialists (A++).

### Management of PTH resistance

Even though PTH resistance is the hallmark of PHP and is referenced in the name of the disorder, very few studies have focused on the natural history of its appearance, and almost none on its management.

Long-term treatment of hypocalcaemia associated with PTH resistance is similar but usually more aggressive than that of primary hypoparathyroidism, with the use of active vitamin D metabolites (calcitriol) or analogues (alfacalcidol) and oral calcium supplements as and when required^179^. The current approach is to reduce the serum level of PTH to the upper portion of the reference range to avoid suppression of PTH, which can be associated with hypercalciuria and renal calcification. Serum levels of PTH that are at the upper limits of the reference range are sufficient to enhance calcium reabsorption in the distal renal tubule, thus helping to prevent hypercalciuria^34^. However, PTH levels should not be too high, as long-standing PTH excess might have adverse effects on skeletal mineralization or on the growth plate^71,180–182^. Accordingly, treatment with oral calcium and active vitamin D supplementation can be used to target a higher serum level of calcium than in patients with PTH-deficient hypocalcaemia, where treatment targets the low–normal or slightly reduced range of calcium. As a result of the preserved sensitivity of the distal convoluted tubules, patients with PHP and related disorders very rarely develop hypercalciuria^34^. However, several experts involved in this Consensus Statement have encountered episodes of nephrolithiasis in patients with PHP1A and PHP1B, particularly after completion of pubertal growth, when the doses of oral calcium and active vitamin D analogues can be reduced (G.M., P.K, A.R. and A.L., unpublished observations).

Chronic hypocalcaemia and associated hyperphosphataemia can result in intracranial deposition of calcium, a feature usually referred to as Fahr syndrome^65^. These calcifications occur predominantly in the basal ganglia but might extend widely to the thalami and the cortex. This form of ectopic calcification is due to elevated levels of the calcium–phosphorus product and hence has not been described in patients with PPHP or POH or those with a mutation in the *PRKAR1A* or *PDE4D* genes^5,6,8,62,183,184^.

Other ectopic depositions of calcium and phosphorus occur in the eyes, which lead to cataracts (peripheral lenticular opacities). Corneal opacities, macular degeneration, nystagmus, anisocoria, papilloedema, tortuosity of retinal vessels and microphthalmia have also been reported^8,185–188^.

Long-term and excessive secondary hyperparathyroidism can result in osteitis fibrosa cystica and slipped capital femoral epiphysis, which might require a combination of medical and surgical management^189,190^. Patients with *GNAS* mutations involving the paternal allele or *PDE4D* mutations usually do not develop PTH resistance over time^15,20,23,60,62,63^.

Additional complications of PTH resistance include dental manifestations, such as failure of tooth eruption, short blunted roots, dental pulp alterations, hypodontia and enamel hypoplasia. Finally, increased uric acid excretion has been reported in two families with PHP1B, which suggests a possible role for PTH resistance in the renal handling of this analyte^191,192^. Increased excretion of uric acid and phosphorus might increase the risk of developing kidney stones if urinary levels of calcium are increased. These last complications, when present, should be managed according to good clinical practice. No specific recommendations can be formulated at the moment given the rarity of their presentation.

***Recommendations***

3.2.   At diagnosis or before initiation of treatment, we recommend the monitoring of serum levels of PTH, calcium, phosphorus and calcifediol. Measurement of PTH, calcium and phosphorus should be performed regularly (every 6 months in children and at least yearly in adults) with the exception of patients carrying either a *GNAS* mutation on the paternal allele or a *PDE4D* mutation in whom, apart from at diagnosis, routine assessment is not necessary. Monitoring of serum levels of calcium should be more frequent in symptomatic individuals, during acute phases of growth, during acute illness and during pregnancy and breastfeeding, when dose requirements for active vitamin D metabolites or analogues might change. Calcifediol levels should be normalized and maintained in the normal range in all patients (A+++).

3.3.   Severe symptomatic hypocalcaemia requires immediate correction, which might include intravenous administration of calcium salts according to general guidelines for the management of acute hypocalcaemia in hypoparathyroidism^179^. These patients should also be concomitantly treated with active vitamin D metabolites or analogues (A+++).

3.4.   In patients with substantial and progressive increases in levels of PTH and hyperphosphataemia, treatment with active vitamin D metabolites or analogues could be considered, independently of the presence of hypocalcaemia. Calcium supplements should be considered, depending on the dietary calcium intake. Serum levels of phosphorus should be monitored during treatment with vitamin D metabolites or analogues and calcium supplements (A++).

3.4.b. Treatment with active vitamin D metabolites or analogues could also be considered when levels of PTH are more than twice the upper level of normal, independently of the presence of hypocalcaemia (B+).

3.5.   The objectives of conventional management of PTH resistance include maintenance of serum levels of calcium and phosphorus within the normal range while avoiding hypercalciuria (age and size corrected) and lowering PTH levels as permitted by serum and urinary levels of calcium. We recommend the use of the active vitamin D metabolite calcitriol or the active vitamin D analogue (alfacalcidol) with or without calcium supplementation as the mainstay of treatment of chronic hypocalcaemia. Patients should not be treated with PTH or PTH analogues. During treatment, levels of PTH, calcium and phosphorus should be monitored every 6 months in asymptomatic patients and more frequently when clinically indicated. Patients and/or their family should be instructed about symptoms and signs of hypocalcaemia and hypercalcaemia (A++).

3.6.   We recommend appropriate renal imaging to evaluate nephrocalcinosis at transition (B+).

3.7.   We recommend age-appropriate^179^ renal imaging to monitor for the development or worsening of nephrocalcinosis in patients with persistent hypercalciuria on repeated measurements and as clinically indicated (B++).

3.8.   For the evaluation of long-term consequences of hypocalcaemia and hyperphosphataemia, a brain CT scan is indicated only when neurological manifestations are present. Ophthalmological examination is recommended to diagnose or exclude cataracts (A++).

3.9.   Phosphate binders (other than calcium) are rarely, if ever, indicated in the management of severe and long-term persistent hyperphosphataemia (B++).

3.10.  We recommend regular dental reviews every 6–12 months during childhood and as clinically indicated in adults (A++).

### Management of TSH resistance

In patients with PHP1A, the average level of TSH is 14.1 ± 10.3 mUI/l, with a range of 1.4 mUI/l to 46.0 mUI/l (refs^24,30,38,67,98,100,117,119,122,123,129,144–146,149,193–205^). A prompt diagnosis of hypothyroidism after birth and initiation of treatment does not seem to prevent the development of motor or cognitive delay^24^.

In patients with PHP1B, the degree of TSH resistance can vary with time^52,53,102,206^. The average level of TSH is 5.3 ± 4.7 mUI/l (4.8 ± 3.4 mUI/l and 5.4 ± 5.2 mUI/l in autosomal dominant PHP1B and sporadic PHP1B, respectively), ranging from 0.8 mUI/l to 50.0 mUI/l (refs^36,44,46–49,51,53,54,87,89,91,102,105,131,143,153,164,166,169,178,200,206–210^).

The literature does not contain specific data on how to treat TSH resistance in patients with PHP and related disorders. Indications for treatment, drug formulations, doses and target serum concentrations of TSH and T_4_ should be the same as those used in any other form of hypothyroidism or subclinical hypothyroidism.

In general, patients should be screened for auto-immune thyroid disease. Nevertheless, because autoimmune thyroid disease is highly prevalent, the presence of autoimmunity will not rule out concomitant TSH resistance in a given patient (see recommendation 3.11).

***Recommendations***

3.11. Evaluation of thyroid function (including autoantibodies in adolescents and adults) for early detection of TSH resistance and early intervention is recommended in all patients with PHP and related disorders at diagnosis. Thereafter, TSH monitoring is recommended every 6 months in patients <5 years of age and yearly in older children and adults (A++).

3.12. The indication or indications to treat hypothyroidism, the dosage of levothyroxine and the therapeutic goals should be the same as for any patient with hypothyroidism or subclinical hypothyroidism^211–213^ (A++).

### Management of growth and GH deficiency

With the exception of PHP1B, a large proportion (around 80% of patients with PHP1A, 50–70% of patients with PPHP and almost all patients with acrodysostosis) of patients with PHP and related disorders have adult short stature^5,13–15,23,24,28,39,45,53,54,62–64,86,89,97,104,105,107,113,122,143,145,146,153,205,206,214–216^. Overall, the final height deficit in patients with PHP1A and PPHP is severe, approximately −2.5 s.d., with mean heights of 149 cm in women and 155 cm in men^28,122,217^. In patients with PHP1B, despite a great variability in adult height, mean stature is not significantly different from that of the general population^107^. Patients with acrodysostosis have adult heights that are even shorter than those of patients with PHP1A, with a mean height of −3.5 s.d. (−8.8 to −0.5)^5,14,15,62–64^. The height deficit in patients with PHP develops over time. Small cohorts of patients with PHP1A have shown declining growth velocity a few years after birth, a lack of a pubertal growth spurt and premature cessation of statural growth^86,97,107,217^. This finding is also supported by observations from the authors’ day-to-day practice (L.d.S., H.J., A.L., P.K., G.M., G.A.M.-M., O.M., A.R. and Su.T., unpublished observations).

Bone age is advanced by more than 2.0 s.d. beyond chronological age in 70–80% of patients with PHP1A^97,215,217,218^. Advanced growth plate maturation is consistently described in patients with acrodysostosis^5,15,63^. The severity of the dysostosis reduces the reliability of using bone age determinations to predict adult height^219^. We know from our practice that phalanx epiphyses might close as early as 3 years of age in these children (A.L., G.A.M.-M., A.R. and Su.T., unpublished observations).

Most patients with PHP1A (50–80%) develop GHRH resistance and consequently GH deficiency^45,86,97,143,146,206,215,218^. Recombinant human growth hormone (rhGH) treatment increased growth velocity in one study conducted in eight prepubertal children with PHP1A and GH deficiency. It is of interest that in this study, one female patient whose oestrogen production was blocked by GnRH analogues did have evidence of a long-term growth advantage compared with her sister who did not receive the analogues^217^. Data from a clinical trial (NCT00209235)^220^ on final height and on the association of puberty blockers are pending.

Finally, in the context of prenatal growth restriction, even if the GH secretion is deficient, the authors’ experience suggests using doses of rhGH higher than those usually given in children with idiopathic GH deficiency, for instance, the doses used in patients with SGA (L.d.S., A.L., G.M. and A.R., unpublished observations). Overall, additional published data are required before a recommendation on the use of GH therapy, or any other growth modifying treatment, in this group of patients can be made.

***Recommendations***

3.13. We recommend careful monitoring of height in children at every control examination (at least every 6 months) until final height is reached (A+++).

3.14. We recommend monitoring of skeletal maturation using plain radiography in all children (with the exception of those with PHP1B) and evaluation for GH deficiency in all children in the context of statural growth deceleration. As most patients develop GH deficiency due to GHRH resistance, clinical and/or biochemical evaluation of the GH–insulin-like growth factor 1 (IGF1) axis should be performed in all patients, typically around the age of 3–6 years and repeated as necessary (B++).

3.15. Patients with GH deficiency should be treated with rhGH. Data are needed on the outcomes of GH treatment in children who are not GH deficient before recommending this treatment in these patients as well (A++).

3.16. Adults with GH deficiency might be considered for treatment with rhGH; however, specific proof of benefit in this population is lacking, and treatment should be given according to country-specific regulations (B++).

3.17. Patients with PHP who are born SGA who do not exhibit appropriate catch-up growth might qualify for treatment with rhGH; however, caution should be exercised in patients with ectopic ossifications, as no data are available on the possible effect of rhGH treatment on the evolution of ossifications (B++).

### Alterations in gonadal function

#### Gonadal function and puberty

The pubertal growth spurt might be blunted or absent in both girls and boys with PHP1A. Although the basis for this defect is unknown, it could relate to insufficient sex steroid production or premature epiphyseal closure^120^. Systematic data are scarcer for PHP1B and PPHP than for PHP1A, but these patients are thought to have normal gonadal function^206^. Similarly to PHP1A, variable resistance to gonadotropins has been described in patients with acrodysostosis and mutations in the *PRKAR1A* gene^15^, as well as in anecdotal reports of single patients with mutations in *PDE4D*^14^. Furthermore, despite basal gonadotropin and sex steroid levels that are within normal limits, menstrual irregularities seem to be common among female patients with PHP1A^120^. According to the experts’ experience, cryptorchidism is highly prevalent in patients with PHP1A, leading to the hypothesis of a possible role of hormone resistance in the pathogenesis of PHP1A (A.L., P.K., Su.T., A.R. and G.M., unpublished observations).

#### Fertility and pregnancy

Unassisted and uneventful pregnancies have been reported in female patients with PHP1A^120,221^ and autosomal dominant PHP1B^44,72,153,160,216^; these pregnancies are more often seen in women with PPHP, who give birth to offspring with PHP1A^24,36,45,117,122,221–223^. In a few cases, either infertility^193^ or the need for the use of an assisted reproductive technique to obtain pregnancy^47,224,225^ have been reported. No data are available on lactation, but there are no specific reports that these patients are unable to lactate.

#### Menopause

No data are available on menopause and its timing in women with PHP and related disorders.

#### Osteoporosis

The prevalence of osteoporosis in patients with PHP and related disorders is unknown. In these patients, bone loss might occur as a result of untreated hypogonadism, long-term excess levels of PTH, GH deficiency and/or the onset of physiological menopause. The physiological action of PTH on bone is mainly to promote bone resorption, but the extent to which PTH signalling in bone is defective in patients with PHP and related disorders is not completely clear. The bone remodelling response to PTH, which is independent of vitamin D action, seems to be intact in these patients^180^, which suggests a possible increased risk of osteoporosis in patients with sustained increased levels of PTH despite treatment with calcium and vitamin D. Nevertheless, bone density seems to be normal to increased in patients with PHP1A, in particular in those who do not have excessive circulating levels of PTH^218^.

***Recommendations***

3.18. Tanner staging of sexual maturation should be performed at regular intervals to monitor pubertal progression in all patients with PHP or related disorders (A+++).

3.19. Testicular descent and location should be assessed in males with PHP or related disorders. Cryptorchidism, when present, should be corrected and managed according to the standard recommendations (A++).

3.20. We do not recommend routine biochemical assessment of the gonadal status unless clinically indicated. Hypogonadism, when present, should be treated with sex hormones following the same standard criteria, doses and follow-up as any other form of hypogonadism (B++).

3.21. After puberty, menstrual history should be collected at each follow-up visit, and biochemical evaluation should be requested in the presence of oligomenorrhoea or amenorrhoea in women and the presence of hypogonadal symptoms in men (B+++).

3.22. In case of infertility, assisted reproductive treatment can be considered according to national guidelines (A+).

3.23. In patients with PHP and related disorders, natural and induced pregnancies should be monitored from an obstetrical point of view in the same way as any other pregnancy. However, dosages of active forms of vitamin D and levothyroxine might have to be adjusted. The possibility of vaginal delivery might be limited as a result of reduced pelvic size and decreased range of motion of the hips due to local ossifications (A+).

3.24. Management of hypocalcaemia and hypothyroidism should follow the current guidelines available for the management of hypoparathyroidism and hypothyroidism during pregnancy. The newborn should be evaluated for levels of TSH, calcium and phosphorus. Lactation is not contraindicated, but close follow-up and clinical monitoring (particularly of weight) of the baby are advised (B++).

3.25. Patients with PHP and related disorders have several potential risk factors for osteoporosis (hypogonadism, chronic elevation of PTH and GH deficiency). However, owing to the lack of evidence of increased fracture risk, there is no indication to perform routine dual-energy X-ray absorptiometry (DXA) measurements in patients with PHP and related disorders. If osteoporosis is diagnosed, management should take into account, whenever possible, treatment of the underlying secondary cause for bone loss (hypogonadism, postmenopausal status or related to sustained elevation of PTH levels and GH deficiency) (B++).

### Management of other hormone resistances

Resistance to additional hormones that mediate their actions through G_s_α-coupled receptors, as well as prolactin deficiency, has also been previously reported; however, the clinical relevance of these abnormalities remains to be established^132^. Early studies described the presence of resistance to calcitonin^114^, and this finding was subsequently confirmed in case reports and in a small case series of patients with PHP1A^115^. Unpublished but consistent observations from the authors indicate that, among the other hormone resistances, hypercalcitoninaemia is present in a substantial subset of patients with PHP1A and PHP1B (L.G., A.L., H.J., G.M., A.R., L.R. and A.H.S., unpublished observations). No published evidence indicates that hypercalcitoninaemia leads to clinically significant C cell hyperplasia or medullary thyroid carcinoma in PHP and related disorders. Adrenal resistance to adrenocorticotropic hormone (ACTH) and other hormone resistances are not documented to occur in patients with PHP1A, apart from a few old anecdotal reports.

***Recommendation***

3.26. We do not recommend routine calcitonin measurement or screening for additional hormone resistances in patients with PHP and related disorders (A+).

### Obesity and other metabolic issues

Children with PHP1A and obesity show both decreased resting energy expenditure compared with controls with obesity and hyperphagic symptoms similar to those seen in BMI-matched controls with obesity^39,111,112^. In older (late infancy, adolescence and young adulthood) patients, this hyperphagic trait seems to abate^111^, and energy expenditure seems to improve to low–normal^113^. As a consequence, obesity is less pronounced in adulthood than in childhood^28^.

With the exceptions of the case reports of a patient with PHP1C successfully treated with a cannabinoid receptor type 1 (CB1) antagonist^99^ and of a patient with PHP1A treated with a gastric bypass^226^, there are no specific reports concerning specialized management of obesity in these patients.

Early-onset obesity is observed not only in patients with PHP1A but also in patients with PHP1B^44,46,86,158^. The increased weight gain might start within the first months of life in some patients, similar to what is observed in patients with monogenic defects of the leptin–melanocortin pathway^227,228^. This finding highlights the need for close monitoring of weight in all patients with PHP and related disorders, not only in those with PHP1A.

Findings from animal models suggest that the underlying mechanism contributing to obesity is related to the effect of G_s_α imprinting in the CNS. In the hypothalamus, G_s_α signals through melanocortin^229^ and thereby affects energy expenditure. This finding suggests that drugs modifying the melanocortin pathway might be effective for weight control in patients with PHP and related disorders.

Sleep apnoea is a well-known complication of obesity. Sleep disturbances with daytime somnolence have been reported in a series of patients with PHP1A^230^ and were more frequent (4.4-fold higher relative risk) than in participants without PHP1A who had similar levels of obesity^230,231^. Irrespective of the underlying mechanism, untreated sleep apnoea is associated with poor memory and concentration, increased risk of heart disease and impaired glucose metabolism^232^. Effective treatment of sleep apnoea might mitigate these risks and even improve school performance^233^. In addition to sleep apnoea, other pulmonary disorders (such as an increased prevalence of asthma) have also been associated with PHP1A (50–75%), PPHP and PHP1B^39,46,230,234^.

Metabolic consequences of PHP and related disorders have not been characterized. Nevertheless, in addition to obesity, impairment of glucose metabolism and hypertension are present in a large subset of patients^112,113^. For instance, patients with PHP1A have decreased insulin sensitivity that appears early during childhood, which seems to be only partially related to the degree of obesity. These data have been published in single case reports^109^, as well as in two small series of children and adolescents (aged 2–18 years)^112,113^, but are also confirmed in daily clinical practice (A.L., G.A.M.-M., A.H.S. and A.R., unpublished observations).

In a series of ten children with PHP1A, levels of HbA_1c_ and fasting insulin were normal in all participants but one^97^. By contrast, in ten adults with PHP1A, four already had a diagnosis of type 2 diabetes mellitus, the fasting plasma levels of glucose and HbA_1c_ were higher than the controls with obesity, and they showed decreased insulin sensitivity^113^. These findings indicate that factors other than obesity might contribute to lower insulin sensitivity in these patients and are consistent with observations in a mouse model of PHP1A in which glucose intolerance and insulin resistance developed before the onset of obesity^235^.

As for the lipid profile in PHP and related disorders, in a small series of patients with PHP1A, cholesterol, triglycerides and/or LDL levels were either elevated or at the upper end of the normal range^97^. No data are available regarding lipid profiles in the other PHP subtypes and related disorders.

Finally, blood pressure and its regulation have been very poorly investigated in PHP and related disorders, and the scarce available data date back to the 1980s^236^. Nevertheless, hypertension is frequently observed in young adults with PHP and related disorders. In 1988, elevated blood pressure was reported in 53% of adult patients with PHP and related disorders, with a similar prevalence in clinically defined patients with PHP1A and PHP1B^237^. More recently, the case of a young patient with PHP1B who had juvenile renin-dependent hypertension has been reported^238^. A direct link between the cAMP signal transduction pathway and hypertension has been demonstrated by the finding of *PDE3A* mutations in families with autosomal dominant hypertension and brachydactyly type E syndrome^21,239^, and the mechanism underlying hypertension in these patients is thought to be related to increased peripheral vascular resistance due to vasoconstriction.

***Recommendations***

3.27. We recommend regular monitoring of BMI and eating behaviour (Table 4). Educational programmes, as well as psychological support, should be provided to patients and families when obesity and/or eating disorders are present and even in the presence of a normal BMI as a preventive strategy, as these patients are at high risk. Dietary counselling should take into account that these patients have decreased resting energy expenditure (A++).

3.28. We recommend that all patients with PHP and related disorders should be evaluated for symptoms such as restless sleep, snoring, inattentiveness and daytime somnolence, and, if symptoms are present, polysomnography is recommended (B++).

3.29. Lipid and glucose metabolism should be monitored on a regular basis (Table 4) (B++).

3.30. Blood pressure should be monitored regularly (Table 4) with an appropriately sized cuff. Patients with hypertension should be treated as clinically indicated (B++).

### Management of ectopic ossifications

In PHP and related disorders, particularly POH, triggering environmental factors for ectopic ossifications, such as trauma, infection and/or metabolic or immune abnormalities, have not been systematically studied^240^; however, clinical experience strongly suggests that these factors do not contribute to new ossifications. By contrast, in FOP, another disease featuring ectopic bone formation, external factors, such as trauma or infections, are well-known triggering events contributing to new bone formation or worsening of existing ones^10^.

A systematic literature search led to the following observations. First, no external factor has been shown to induce the development of ectopic ossifications in patients with PHP1A, PPHP or POH. Second, no correlation exists between PTH levels or serum levels of calcium–phosphorus and the occurrence of ectopic ossifications. Third, ectopic ossifications often occur in locations subjected to high pressure loads, such as the heel^81^. Fourth, plate-like or severe ossifications might develop within the first year of life and progressively invade the deep tissues^58^. Finally, paternally inherited mutations at *GNAS*, mainly those leading to a truncated protein, have been reported as contributing to the development of ectopic ossifications^41,145^.

As a result of the rareness of these conditions, limited information is available about prognosis. Currently, no effective treatments exist for the management or prevention of ectopic ossifications. Surgical removal of the ectopic ossifications can be performed when the lesions are well delimited. This is rarely the case in patients with POH, but it can be effective in those with osteoma cutis and in superficial lesions associated with PPHP or PHP1A with successful long-term results^58,59,81,241^. In most cases, surgical resection is followed by recurrence or complications^3,83,242–245^. Successful functional repositioning of a joint after the development of a contracture due to ectopic ossifications was reported in one child^243^. Amputations are sometimes needed in the setting of severe growth retardation and functional ankyloses^83^ or following the development of severe infections in recurrent skin ulcerations^242^.

Bisphosphonates have been proposed to prevent the post-surgical complications and recurrences of ossifications owing to their known effect on inhibiting bone turnover. Although we are lacking data from controlled trials, in five patients with neurogenic or post-traumatic ectopic ossifications, pamidronate seemed to prevent recurrences^79^. Pamidronate has also been used in a single patient with POH as a primary treatment of progressing heterotopic ossifications, with an apparent delay in new bone formation, rather than a change in the ectopic bone itself^246^. Etidronate has also been used with an apparent beneficial effect on the progression of ectopic ossifications in one patient with a POH-like presentation^247^, but no improvement was reported in another patient with POH^248^. Finally, topical sodium thiosulfate has been successfully administered in patients with hyperphosphataemic familial tumoural calcinosis or those with hyperphosphataemia–hyperostosis syndrome with a clinically and radiologically significant decrease of ectopic ossifications^249^. Preliminary data indicate that bisphosphonates might also delay the progression of ectopic ossifications in patients with ossifications secondary to PHP or related disorders^250^. Physical therapy and meticulous skin care remain the most important conservative approaches to preserve movement and to prevent cutaneous breakdown, respectively^251,252^.

***Recommendations***

3.31. The presence of cutaneous bony plaques should be investigated by careful examination at each visit in all patients with *GNAS* mutations, especially those with mutations on the paternal allele (POH and PPHP). Patients and families should be instructed about self-examination. We recommend documenting the following at each visit: location and size of ossifications; involvement of joints and impairment of movement and bone growth; predilection of lesions towards areas exposed to increased pressure due to weight bearing (feet and ankles); assessment of triggering events (trauma, infection, inflammation and surgery); association with pubertal development; and treatment with rhGH (A++).

3.32. Regular imaging of ectopic ossifications is not indicated (A++).

3.33. Imaging of ossifications should be performed using CT or MRI, depending on localization, when the lesions are painful, symptomatic, jeopardize joint or organ function or are being considered for surgical excision (A++).

3.34. When the diagnosis underlying ectopic ossifications is doubtful and when FOP has been excluded, there is no contraindication to perform a biopsy of the lesion, as no evidence suggests that inflammation or trauma leads to progression of ectopic ossifications in PHP and related disorders (B++).

3.35. Physical therapy and meticulous skin care are the most important approaches for the prevention of development and/or progression of ectopic ossifications. Surgical excision should be considered in the presence of delimited, superficial lesions associated with pain and/or movement impairment. The patient should be referred to a surgeon with experience in the management of ectopic ossifications. In extensive ossifications around a joint, avoid immobilization (through casts), as this might lead to ankyloses of the joint (B+).

3.36. There is no evidence for recommending the use of nonsteroidal anti-inflammatory drugs, bisphosphonates or steroids in primary or peri-surgical treatment of asymptomatic ectopic ossifications (B+).

### Brachydactyly and orthopaedic issues

#### Skeletal complications

Brachydactyly might contribute to difficulty with fine motor skills, such as handwriting, in children with PHP1A, leading to the need for occupational therapy services in early childhood^253^. A high incidence of carpal tunnel syndrome has been observed in patients with PHP1A and PPHP (67% of patients with PHP1A reported symptoms versus 15% of the general population)^253^.

Additional skeletal features have been described in different disorders of the PTH–PTHrP pathway, such as Madelung deformity^72,128^, spinal stenosis^203,254–257^, acro-osteolysis, cortical irregularity of long bones and metadiaphyseal enchondromata or short humerus and curved radius^258^, as well as other craniofacial peculiarities (typical pear-shaped nose, long and flat philtrum, thin upper lip and receding chin) and phalangeal cone-shaped epiphyses, resulting in clinodactyly^259^. Depending on the functional consequences, the patient might require corrective orthopaedic surgery.

Finally, many paediatric patients have a history of recurrent otitis media requiring a tympanostomy tube or tympanoplasty^230^.

#### Oral complications

Several oral manifestations have been described in patients with PHP and related disorders, including aplasia, thin enamel with enlarged pulp chamber, hypoplasia, hypodontia, pulp calcification, multiple carious teeth, multiple unerupted teeth or delayed tooth eruption, crowded anterior teeth, anterior open bite, gingival hyperplasia, gingivitis with spontaneous bleeding and pain^88,260^. Familial nonsyndromic primary failure of tooth eruption has also been described in patients with mutations in *PTHR1* and *PTHrP*^88,261^.

***Recommendations***

3.37. In addition to their use for diagnosis and characterization of brachydactyly, further specific radiological investigations should be reserved for patients with specific clinical suspicion of orthopaedic malformation or functional impairment, particularly in the presence of neurological signs or symptoms. Patients with severe brachydactyly should receive formal evaluation of their fine motor skills as well as be supplied with appropriate orthopaedic devices when indicated (that is, special shoes and orthopaedic insoles) (A++).

3.38. Specific multidisciplinary evaluation and therapy should be offered for rare orthopaedic or neurological manifestations: spinal stenosis, bilateral slipped capital femoral epiphyses, temporomandibular joint ankyloses, precocious scoliosis, Chiari malformation type 1 and cranial synostosis (A++).

### Management of cognitive function

Patients with PHP1A present with psychomotor and cognitive abnormalities, which are defined as a history of developmental delay and learning disability, with reduced performance on the Wechsler intelligence scale^24,42,262^ and an increased incidence of psychiatric manifestations that might be the consequence of the disease or of long-term hypocalcaemia^42,207^.

In general, global retardation of developmental milestones, psychomotor retardation, delayed speech or the need for an assistant teacher and extra school help are reported^18,263,264^. The performance IQ is more affected than the verbal IQ^24^. Intellectual disability seems to be more prevalent in patients with PHP1A than in patients with PPHP, which suggests that G_s_α is imprinted in the brain^24,214,229^. The existence of neurological manifestations or true neuropsychiatric phenotype due to organic CNS alterations, and specifically Chiari malformation type 1, should be considered^38,207,265^. Finally, the prevalence and severity of cognitive impairment and developmental delay vary among patients with acrodysostosis, but a detailed clinical description is lacking^5,6,14,63,266^.

***Recommendation***

3.39. Consider referral to a neuropsychologist for neurocognitive and/or behavioural assessment at diagnosis or at preschool age, particularly in patients with PHP1A and acrodysostosis due to *PDE4D* mutations, and also if otherwise appropriate (for instance, if the patient presents with symptoms of cognitive impairment). Additional testing and support as required should also be considered (A++).

### Malignancy risk

Three case reports have described the coexistence of PHP or a related disorder with malignancy: PHP1A and cerebellar pilocytic astrocytoma^267^, PHP1B and osteosarcoma^168^ and POH with medulloblastoma^82^. However, large-scale studies aimed at determining tumour risk in PHP and related disorders have not been carried out. A Danish study has investigated all patients with a diagnosis of PHP or a related disorder (clinical and/or genetically confirmed) to determine their mortality data and risk of complications using data from the Danish National Patient Registry^8^. With a total of 60 cases, patients with PHP or a related disorder were found to have an increased risk of neuropsychiatric disorders, infections, seizures and cataracts, whereas their risk of renal, cardiovascular and malignant disorders and fractures was similar to that of the general background population. The same results were obtained when analysing only the subgroup with genetically verified PHP or a related disorder.

An increased tumour risk might be hypothesized to exist in patients with PHP1B and MLID, mostly patients with MLID that are associated with Beckwith–Wiedemann syndrome, a growth disorder characterized by embryonal tumours. However, the few data collected to date did not report any tumours in patients with PHP1B and MLID^92,104^.

***Recommendation***

3.40. There are no data to recommend a specific screening for malignancies in PHP and related disorders (A+++).

## Conclusions

Patients with PHP and related disorders face a wide range of problems from early childhood to adulthood. These include potentially severe alterations in mineral metabolism, which could be associated with seizures, other endocrine deficiencies due to hormone resistance that lead to hypothyroidism, hypogonadism and GH deficiency, growth impairment independently of hormonal status, ectopic ossifications with potential severe limitation of mobility, skeletal issues and cognitive and psychomotor impairment. This highly heterogeneous clinical picture renders a multidisciplinary approach mandatory in these disorders, as very specialized expertise is required to manage each of the many clinical aspects and potential complications of PHP and related disorders.

In addition, this group of disorders is caused by different and complex genetic and epigenetic defects, such that establishing a correct molecular diagnosis might be difficult and time consuming for both patients and their families and physicians. There is an urgent need to gather forces and cohorts of patients to implement registries, to improve knowledge on the natural history of the diseases, to better understand the bridges between these clinically heterogeneous but still closely related diseases and, moreover, to develop new therapies.

This article, which is based on published evidence and expert opinion, is the first international consensus statement for the diagnosis and management of PHP and related disorders. The 67 recommendations apply to all patients with a clinical diagnosis, independently of molecular confirmation. Nevertheless, in some patients, the identification of the underlying genetic or epigenetic defect might help clinicians to look for specific clinical manifestations with consequent appropriate management. A multidisciplinary approach is needed in most cases, and Table 4 summarizes the main interventions as well as their timing.

Given the lack of strong evidence-based data, particularly for management of these patients, international collaboration and long-term clinical trials looking at the natural history, the diseases’ classification and the outcome of treatments are urgently needed (Box 1).

Box 1 Future research directions **Diagnosis and natural history of the disorders**

- Extensive and systematic data on the following:

- Growth pattern and final heights
- Bone maturation and timing of premature closure of the epiphysis
- Pubertal development and gonadal function (including menopause), fertility and mineralization status
- Metabolic alterations and blood pressure
- Objective evaluation by standardized tests for cognitive and psychomotor impairment
- Identification of factors that contribute to the development and progression of ectopic bone formation
- Pursuit of initiatives for the development of a novel diagnostic classification

**Molecular diagnosis**

- Frequency and associated phenotypes of the different molecular alterations
- Development of testing methodology and validation of technologies for the detection of partial methylation defects
- Prevalence of multilocus imprinting disturbance (MLID) and its effect on the phenotype
- Identification of the prevalence and effects of genetic variants underlying methylation defects (affecting *GNAS* alone or MLID)
- Identification of the prevalence of molecular defects at the parathyroid hormone (PTH)–parathyroid hormone-related protein (PTHrP) signalling cascade in a mosaic state
- Identification of new genes involved in the PTH–PTHrP signalling cascade and their association with as-yet-unresolved cases with PHP and related disorders
- Identification of the receptor pathway upstream of GNAS that is associated with the development of ectopic ossification

**Follow-up and management**

- Optimal calcium, phosphorus and PTH levels during active treatment for PTH resistance
- Complications related to chronic hypocalcaemia, long-term PTH elevation and their treatment
- Indications, doses, efficacy and optimal timing for recombinant human growth hormone (rhGH) use and indication for puberty blockers
- Contribution of reduced energy expenditure, decreased lipolysis, growth hormone-releasing hormone (GHRH) resistance and hyperphagia to the onset and degree of obesity
- Preclinical and clinical trials to investigate the benefit of post-surgical bisphosphonates and of other candidate drugs (inhibitors of the Hedgehog signalling pathway and retinoic acid receptor-γ agonists)
- Quality of life (QoL): both systematic evaluation with validated QoL questionnaires (that is, the 36-item Short Form Survey (SF-36)) and validation of disease-specific questionnaires are needed

## Supplementary information

Supplementary Figure 1 and Table 1
