# Supplementary material for: Diagnosis and management of pseudohypoparathyroidism and related disorders: first international Consensus Statement
Source: Nat Rev Endocrinol. 2018 Jun 29;14(8):476–500. doi: 10.1038/s41574-018-0042-0 (PMC6541219; doi:10.1038/s41574-018-0042-0)
Supplement: Supplementary file 1 — Supplementary Figure 1 and Table 1 [file 41574_2018_42_MOESM1_ESM.pdf]

nature REVIEWS ENDOCRINOLOGY

SUPPLEMENTARY INFORMATION

In format as provided by the authors

Diagnosis and management of pseudohypoparathyroidism and related disorders: first international Consensus Statement

Giovanna Mantovani, Murat Bastepe, David Monk, Luisa de Sanctis, Susanne Thiele, Alessia Usardi, S. Faisal Ahmed, Roberto Bufo, Timothée Choplin, Gianpaolo De Filippo, Guillemette Devernois, Thomas Eggermann, Francesca M. Elli, Kathleen Freson, Aurora García Ramirez, Emily L. Germain-Lee, Lionel Groussin, Neveen Hamdy, Patrick Hanna, Olaf Hiort, Harald Jüppner, Peter Kamenický, Nina Knight, Marie-Laure Kottler, Elvire Le Norcy, Beatriz Lecumberri, Michael A. Levine, Outi Mäkitie, Regina Martin, Gabriel Ángel Martos-Moreno, Masanori Minagawa, Philip Murray, Arrate Pereda, Robert Pignolo, Lars Rejnmark, Rebecca Rodado, Anya Rothenbuhler, Vrinda Saraff, Ashley H. Shoemaker, Eileen M. Shore, Caroline Silve, Serap Turan, Philip Woods, M. Carola Zillikens, Guiomar Perez de Nanclares and Agnès Linglart

https://doi.org/10.1038/s41574-018-0042-0

© 2018 Macmillan Publishers Limited, part of Springer Nature. All rights reserved.

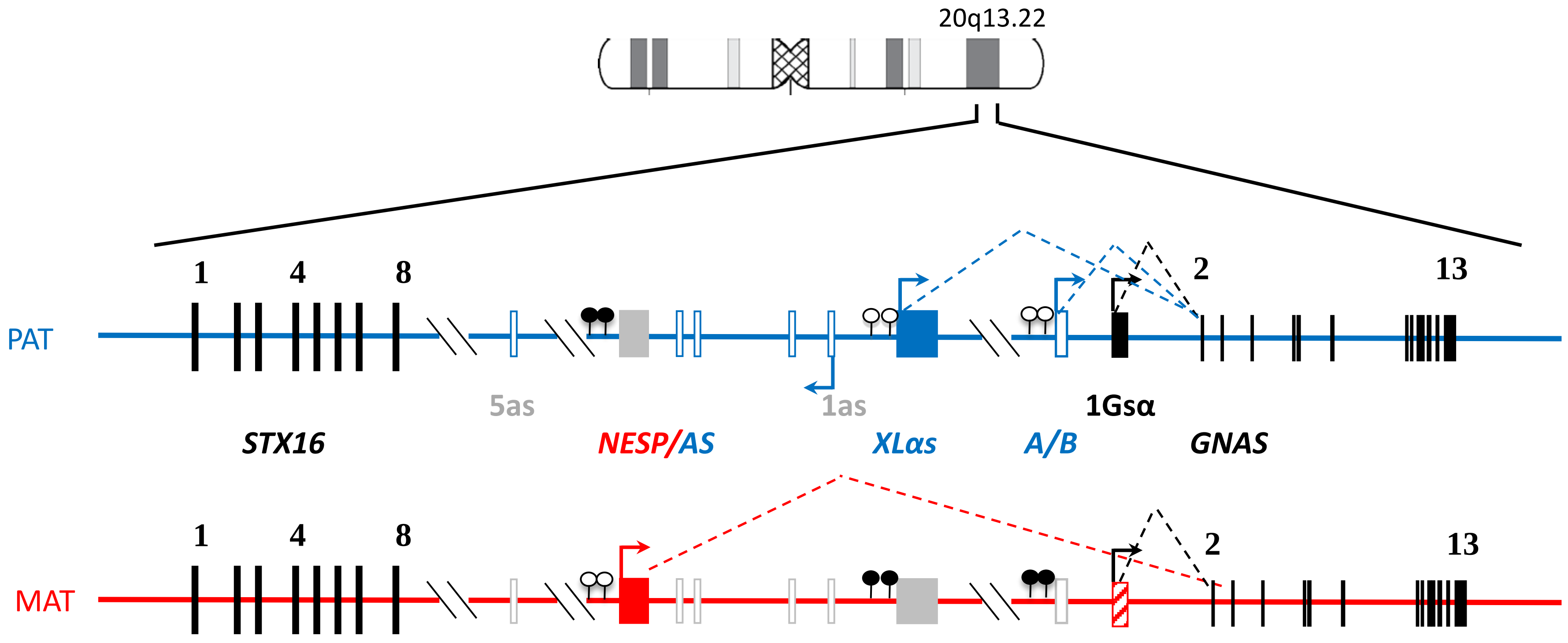

Sup Figure 1A: Scheme of the GNAS locus. The *GNAS* locus has a complex organisation. The shows the normal situation of this imprinted region on the paternal (blue) and the maternal (red) chromosomes. Filled boxes, protein coding genes; empty boxes, non-coding genes; filled lollipops, methylated regions; empty lollipops, unmethylated regions; black, genes with biparental expression; red, genes expressed from the maternal (mat) chromosome; blue, genes expressed from the paternal (pat) chromosome. First exon of Gsα gene in the maternal allele is drawn with hatched red lines to indicate that even biallelically expressed in most tissues, there is a preferable expression of the maternal allele in some specific ones. Arrows above the genes, transcription direction of sense genes; arrows below the genes, transcription direction of anti-sense genes; dotted lines show the splicing of the different genes/exons; double slashes represent the chromosome regions not shown. Adapted with permission from ref. [34], CC-BY-4.0.

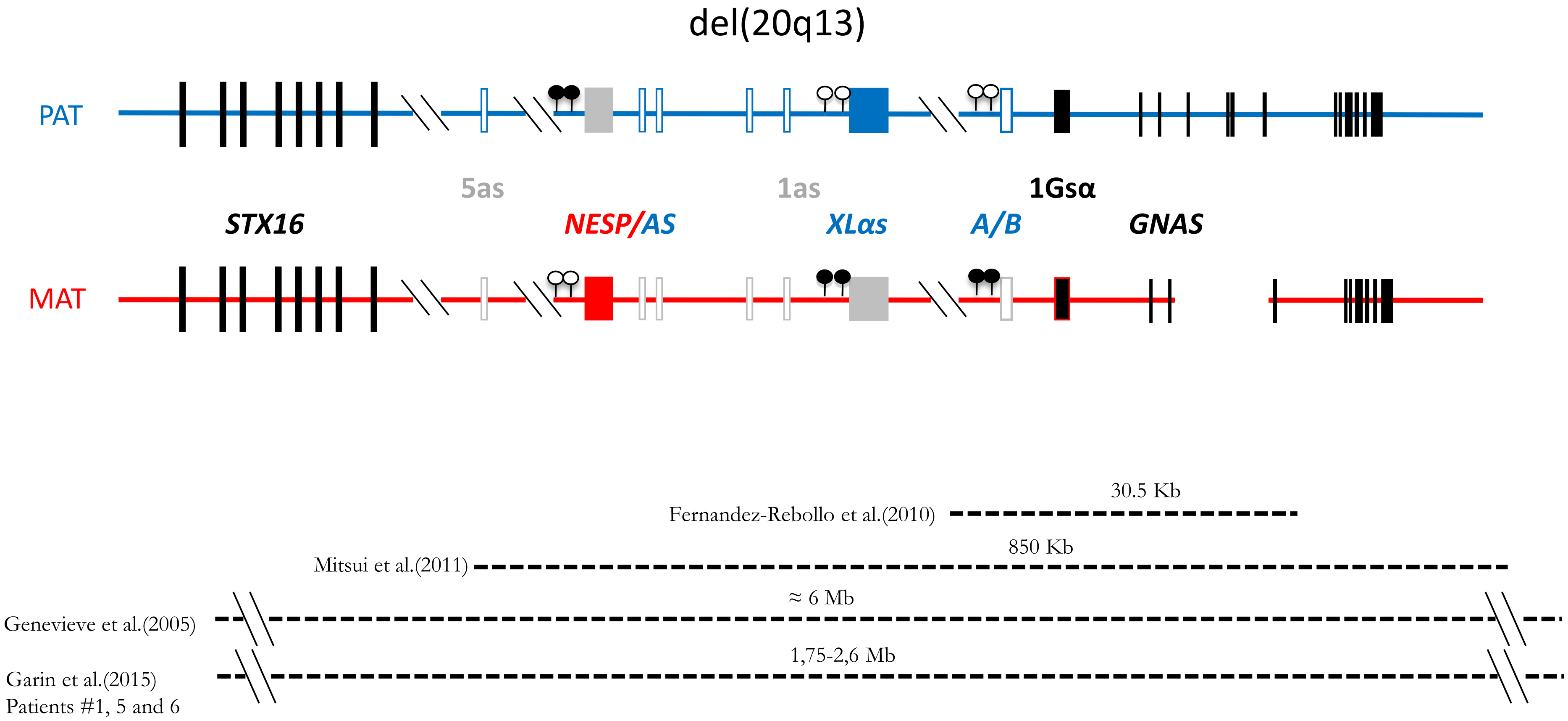

Sup Figure 1B: Genomic rearrangements associated with Pseudohypoparathyroidism type 1A and PPHP. PHP1A can be caused either by point mutations at, or by deletions of, one or more exons of the maternal copy of *GNAS* gene. When the affected allele in the paternal one, PPHP or POH is the clinical outcome. Gross deletions affecting some or all the DMRs of GNAS locus leads to an apparent methylation defect. Reported genetic deletions (spotted black lines) included**.** Adapted with permission from ref. [34], CC-BY-4.0.

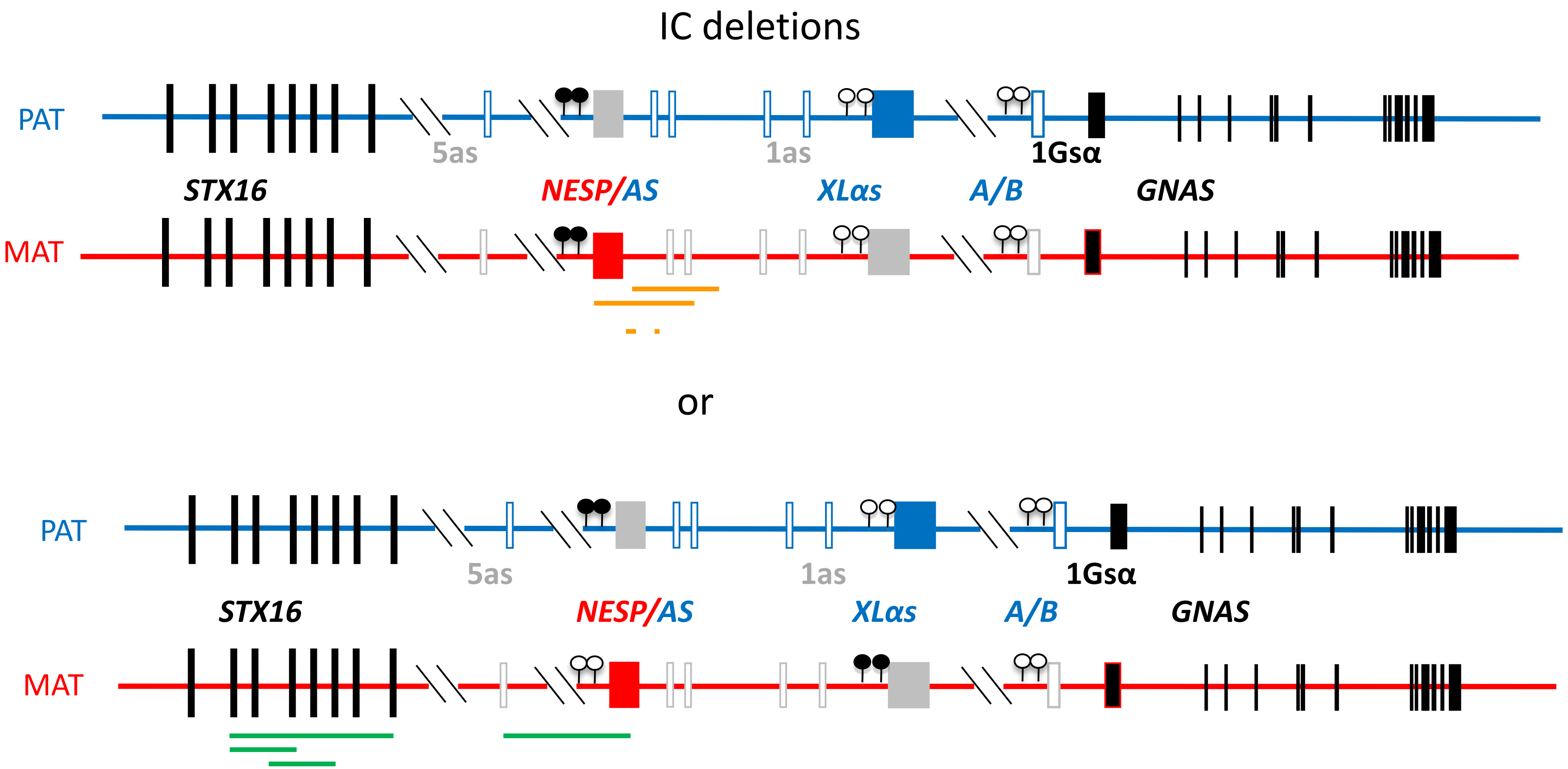

Sup Figure 1C: Genetic and epigenetic variants associated to autosomal dominant pseudohypoparathyroidism type 1B. AD-PHP1B is mostly caused by an isolated imprinting defect at *GNAS A/B:*TSS-DMR (A/B) associated to a deletion at the maternal allele affecting STX16 and NESP55 (green line) or by and overall imprinting alteration at the four DMRs of GNAS locus due to maternal deletions at exons 3 and 4 of GNAS-AS as well as the 40 and 33bp microdeletions at introns 4 and 3 of GNAS-AS (orange lines). Adapted with permission from ref. [34], CC-BY-4.0.

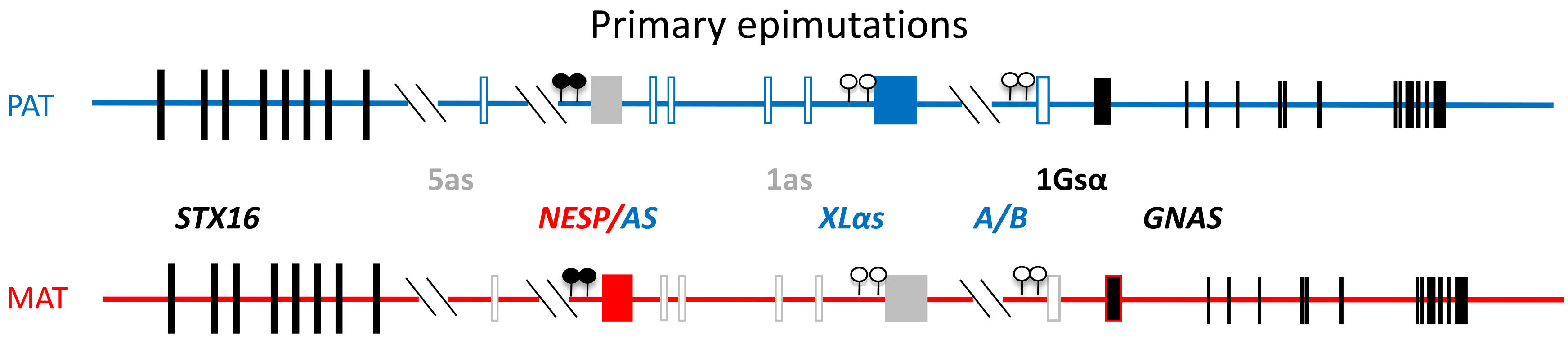

Sup Figure 1D: Epigenetic alterations in sporadic PHP1B. Most PHP1B patients carry methylation defects at two or more DMRs of *GNAS* locus including *GNAS A/B:*TSS-DMR without an identified genetic defect. It this situation a primary epimutation is suspected. Adapted with permission from ref. [34], CC-BY-4.0.

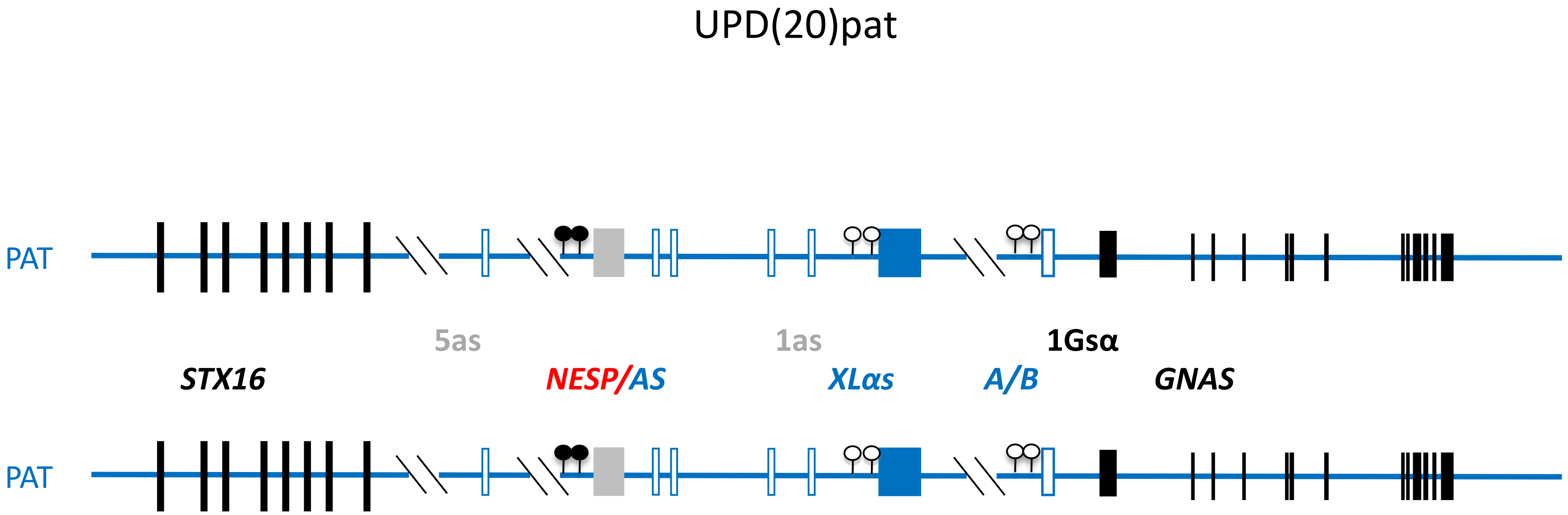

Sup Figure 1E: Uniparental disomy in PHP1B. In around 10% of PHP1B patients the methylation defect is due to a paternal unipaternal disomy (UPD) of chromosome 20q, including the GNAS locus. UPD means that both chromosomal copies have been inherited from the same progenitor. Adapted with permission from ref. [34], CC-BY-4.0.

SUPPLEMENTARY MATERIAL

Supplementary table 1. Molecular defects found associated to the different clinical diagnosis revealing the molecular overlap of PHP and related disorders. Percentages have been obtained from references including case series.*Once inactivating pathogenic mutations at *GNAS* have been excluded

	GNAS		***PRKAR1A***	***PDE4D***
	Genetic defect	Epigenetic defect		
**PHP1A**	70-80%[1–15]	21-60%*[6,16,17]	10%*[4]	8*[4]
**PHP1C**	2-18%[10,18]	20%[19]		
**PPHP**	0-80%[1–3,10,12,15,20]		2*[4]	15%*[4]
**POH**	60-70%[1,9,15,21,22]	0%	0%	0%
**PHP1B**	5-10%[2,3,6,13,17,23–25]	90%[2,3,6,13,17,23–25]		
**Acrodysostosis**	Some cases[26]		50-90%[27–31]	12-40%[27–29,32,33]

REFERENCES

1. Adegbite, N. S., Xu, M., Kaplan, F. S., Shore, E. M. & Pignolo, R. J. Diagnostic and mutational spectrum of progressive osseous heteroplasia (POH) and other forms of GNAS-based heterotopic ossification. *Am. J. Med. Genet. A.* **146A,** 1788–1796 (2008).
2. Cho, S. Y. *et al.* Clinical characterization and molecular classification of 12 Korean patients with pseudohypoparathyroidism and pseudopseudohypoparathyroidism. *Exp. Clin. Endocrinol. Diabetes* **121,** 539–545 (2013).
3. de Sanctis, L. *et al.* Genetic and epigenetic alterations in the GNAS locus and clinical consequences in Pseudohypoparathyroidism: Italian common healthcare pathways adoption. *Ital. J. Pediatr.* **42,** 101 (2016).
4. Elli, F. M. *et al.* Screening of PRKAR1A and PDE4D in a Large Italian Series of Patients Clinically Diagnosed with Albright Hereditary Osteodystrophy and/or Pseudohypoparathyroidism. *J. Bone Miner. Res.* **31,** 1215–24 (2016).
5. Elli, F. M. *et al.* Pseudohypoparathyroidism type Ia and pseudo-pseudohypoparathyroidism: the growing spectrum of GNAS inactivating mutations. *Hum. Mutat.* **34,** 411–416 (2013).
6. Fernández-Rebollo, E. *et al.* Endocrine profile and phenotype-(epi)genotype correlation in Spanish patients with pseudohypoparathyroidism. *J. Clin. Endocrinol. Metab.* **98,** E996-1006 (2013).
7. Garin, I. *et al.* Novel microdeletions affecting the GNAS locus in pseudohypoparathyroidism: characterization of the underlying mechanisms. *J. Clin. Endocrinol. Metab.* **100,** E681-687 (2015).
8. Germain-Lee, E. L., Groman, J., Crane, J. L., Jan de Beur, S. M. & Levine, M. A. Growth hormone deficiency in pseudohypoparathyroidism type 1a: another manifestation of multihormone resistance. *J. Clin. Endocrinol. Metab.* **88,** 4059–4069 (2003).
9. Lebrun, M. *et al.* Progressive osseous heteroplasia: a model for the imprinting effects of GNAS inactivating mutations in humans. *J. Clin. Endocrinol. Metab.* **95,** 3028–3038 (2010).
10. Linglart, A. *et al.* GNAS1 lesions in pseudohypoparathyroidism Ia and Ic: genotype phenotype relationship and evidence of the maternal transmission of the hormonal resistance. *J. Clin. Endocrinol. Metab.* **87,** 189–197 (2002).
11. Mantovani, G. *et al.* Growth hormone-releasing hormone resistance in pseudohypoparathyroidism type ia: new evidence for imprinting of the Gs alpha gene. *J. Clin. Endocrinol. Metab.* **88,** 4070–4074 (2003).
12. Miric, A., Vechio, J. D. & Levine, M. A. Heterogeneous mutations in the gene encoding the alpha-subunit of the stimulatory G protein of adenylyl cyclase in Albright hereditary osteodystrophy. *J. Clin. Endocrinol. Metab.* **76,** 1560–1568 (1993).
13. Reis, M. T. A., Matias, D. T., Faria, M. E. J. de & Martin, R. M. Failure of tooth eruption and brachydactyly in pseudohypoparathyroidism are not related to plasma parathyroid hormone-related protein levels. *Bone* **85,** 138–141 (2016).
14. Shoemaker, A. H. *et al.* Energy expenditure in obese children with pseudohypoparathyroidism type 1a. *Int. J. Obes. 2005* **37,** 1147–1153 (2013).
15. Thiele, S. *et al.* A positive genotype-phenotype correlation in a large cohort of patients with Pseudohypoparathyroidism Type Ia and Pseudo-pseudohypoparathyroidism and 33 newly identified mutations in the GNAS gene. *Mol. Genet. Genomic Med.* **3,** 111–120 (2015).
16. Mantovani, G. *et al.* Pseudohypoparathyroidism and GNAS epigenetic defects: clinical evaluation of albright hereditary osteodystrophy and molecular analysis in 40 patients. *J. Clin. Endocrinol. Metab.* **95,** 651–658 (2010).
17. Elli, F. M. *et al.* Quantitative analysis of methylation defects and correlation with clinical characteristics in patients with pseudohypoparathyroidism type I and GNAS epigenetic alterations. *J. Clin. Endocrinol. Metab.* **99,** E508-517 (2014).

18. Thiele, S. *et al.* Functional characterization of GNAS mutations found in patients with pseudohypoparathyroidism type Ic defines a new subgroup of pseudohypoparathyroidism affecting selectively Gsα-receptor interaction. *Hum. Mutat.* **32,** 653–660 (2011).
19. Brix, B. *et al.* Different pattern of epigenetic changes of the GNAS gene locus in patients with pseudohypoparathyroidism type Ic confirm the heterogeneity of underlying pathomechanisms in this subgroup of pseudohypoparathyroidism and the demand for a new classification of GNAS-related disorders. *J. Clin. Endocrinol. Metab.* **99,** E1564-1570 (2014).
20. Gelfand, I. M., Eugster, E. A. & DiMeglio, L. A. Presentation and clinical progression of pseudohypoparathyroidism with multi-hormone resistance and Albright hereditary osteodystrophy: a case series. *J. Pediatr.* **149,** 877–880 (2006).
21. Shore, E. M. *et al.* Paternally inherited inactivating mutations of the GNAS1 gene in progressive osseous heteroplasia. *N. Engl. J. Med.* **346,** 99–106 (2002).
22. Elli, F. M. *et al.* Screening for GNAS genetic and epigenetic alterations in progressive osseous heteroplasia: first Italian series. *Bone* **56,** 276–280 (2013).
23. Molinaro, A. *et al.* TSH elevations as the first laboratory evidence for pseudohypoparathyroidism type Ib (PHP-Ib). *J. Bone Miner. Res.* **30,** 906–912 (2015).
24. Takatani, R. *et al.* Analysis of Multiple Families with Single Individuals Affected by Pseudohypoparathyroidism Type Ib (PHP1B) Reveals Only One Novel Maternally Inherited GNAS Deletion. *J. Bone Miner. Res.* **31,** 796–805 (2015).
25. Yuno, A. *et al.* Genetic and epigenetic states of the GNAS complex in pseudohypoparathyroidism type Ib using methylation-specific multiplex ligation-dependent probe amplification assay. *Eur. J. Endocrinol.* **168,** 169–175 (2013).
26. Mitsui, T. *et al.* Acroscyphodysplasia as a phenotypic variation of pseudohypoparathyroidism and acrodysostosis type 2. *Am. J. Med. Genet. A.* **164A,** 2529–2534 (2014).
27. Kaname, T. *et al.* Heterozygous mutations in cyclic AMP phosphodiesterase-4D (PDE4D) and protein kinase A (PKA) provide new insights into the molecular pathology of acrodysostosis. *Cell. Signal.* **26,** 2446–2459 (2014).
28. Linglart, A. *et al.* PRKAR1A and PDE4D Mutations Cause Acrodysostosis but Two Distinct Syndromes with or without GPCR-Signaling Hormone Resistance. *J. Clin. Endocrinol. Metab.* **97,** E2328–E2338 (2012).
29. Lynch, D. C. *et al.* Identification of Novel Mutations Confirms PDE4D as a Major Gene Causing Acrodysostosis. *Hum. Mutat.* **34,** 97–102 (2013).
30. Muhn, F. *et al.* Novel mutations of the PRKAR1A gene in patients with acrodysostosis. *Clin. Genet.* **84,** 531–538 (2013).
31. Linglart, A. *et al.* Recurrent PRKAR1A mutation in acrodysostosis with hormone resistance. *N. Engl. J. Med.* **364,** 2218–2226 (2011).
32. Lee, H. *et al.* Exome Sequencing Identifies PDE4D Mutations in Acrodysostosis. *Am. J. Hum. Genet.* **90,** 746–751 (2012).
33. Michot, C. *et al.* Exome sequencing identifies PDE4D mutations as another cause of acrodysostosis. *Am. J. Hum. Genet.* **90,** 740–745 (2012).
34. Eggermann, T, *et al.* Imprinting disorders: a group of congenital disorders with overlapping patterns of molecular changes affecting imprinted loci. *Clin. Epigenetics*. **7,** 123. doi: 10.1186/s13148-015-0143-8 (2015)
